# DYRK1B mutations associated with metabolic syndrome impair the chaperone-dependent maturation of the kinase domain

**DOI:** 10.1038/s41598-017-06874-w

**Published:** 2017-07-25

**Authors:** Samira Abu Jhaisha, Esti W. Widowati, Isao Kii, Rie Sonamoto, Stefan Knapp, Chrisovalantis Papadopoulos, Walter Becker

**Affiliations:** 1 Institute of Pharmacology and Toxicology, RWTH Aachen University, Aachen, Germany; 2 Chemistry Study Program, Faculty of Science and Technology, State Islamic University, (UIN), Sunan Kalijaga, Yogyakarta Indonesia; 3 Pathophysiological and Health Science Team, Imaging Platform and Innovation Group, Division of Bio-Function Dynamics Imaging, RIKEN Center for Life Science Technologies, 6-7-3 Minatojima-minamimachi, Chuo-ku, Kobe 650-0047 Japan; 4 Institute for Pharmaceutical Chemistry and Buchmann Institute for Molecular Life Sciences (BMLS), Johann Wolfgang Goethe University, Frankfurt am, Main 60438 Germany; 5 Present Address: Molecular Biology I, Center for Medical Biotechnology, University of Duisburg-Essen, Essen, Germany

## Abstract

Two missense mutations of the *DYRK1B* gene have recently been found to co-segregate with a rare autosomal-dominant form of metabolic syndrome. This gene encodes a member of the DYRK family of protein kinases, which depend on tyrosine autophosphorylation to acquire the catalytically active conformation. The mutations (H90P and R102C) affect a structural element named DYRK homology (DH) box and did not directly interfere with the conformation of the catalytic domain in a structural model of DYRK1B. Cellular assays showed that the mutations did not alter the specific activity of mature kinase molecules. However, a significant part of the mutant DYRK1B protein accumulated in detergent-insoluble cytoplasmic aggregates and was underphosphorylated on tyrosine. The mutant DYRK1B variants were more vulnerable to the HSP90 inhibitor ganetespib and showed enhanced binding to the co-chaperone CDC37 as compared to wild type DYRK1B. These results support the hypothesis that the mutations in the DH box interfere with the maturation of DYRK1B by tyrosine autophosphorylation and compromise the conformational stability of the catalytic domain, which renders the kinase susceptible to misfolding.

## Introduction

DYRK1B (dual-specificity tyrosine(Y)-phosphorylation regulated kinase 1B) is a member of the DYRK family of protein kinases that attain full catalytic activity by co-translational autophosphorylation on a conserved tyrosine residue in the activation loop of the catalytic domain^1–3^. DYRK1B is overexpressed in certain cancers and has been characterized as a regulator of cell differentiation and cell cycle progression^4–6^.

Two missense mutations of the *DYRK1B* gene, H90P and R102C, were recently found to co-segregate with a rare autosomal-dominant form of metabolic syndrome called AOMS3 (abdominal obesity-metabolic syndrome 3, OMIM entry #615812). Affected individuals develop early-onset coronary artery disease, hypertension, central obesity, and diabetes^7^. This important discovery shows that a single cause can trigger the full phenotype of metabolic syndrome. Based on the results of cell-based assays, the mutant alleles were proposed to encode gain-of-function variants of DYRK1B^7^. Overexpression of DYRK1B-H90P or DYRK1B-R102C in HepG2 hepatoma cells resulted in a higher induction of the key gluconeogenic enzyme, glucose-6-phosphatase (G6Pase), than overexpression of wild type DYRK1B. Furthermore, the R102C mutation potentiated the effect of DYRK1B on the adipogenic differentiation of 3T3-L1 preadipocytes^7^. How the mutations alter the molecular function of DYRK1B in these assays remains unknown.

In contrast to the gain-of-function effects in these cellular assays, DYRK1B-R102C was found to have reduced catalytic activity in *in vitro*-kinase assays^6^, while the effect of the H90P mutation on kinase activity has not yet been studied. Interestingly, H90 and R102 are located N-terminal of the catalytic domain and are unlikely to be directly involved in substrate recognition or catalysis. A nuclear localization signal and a binding site for the adaptor protein DCAF7^8^ are located N-terminal of the mutated sites.

Maturation of most protein kinases depends on the collaborative action of HSP90 and the kinase-specific co-chaperone CDC37^9^. Recent findings indicate that the interaction of a kinase with HSP90/CDC37 is not determined by particular sequence motifs, but rather by the conformational status of the catalytic domain^10, 11^. In fact, the strength of the interactions between different kinases and HSP90/CDC37 correlated with the thermal instability of the kinase domain. Point mutations can increase or decrease HSP90 interactions with individual kinases in correlation with their effects on the thermodynamic stability of the kinase folds^10, 12, 13^. By buffering destabilizing mutations, the HSP90 system allows its clients to tolerate genetic variation, which may be a key factor in the expansion of the protein kinase superfamily^14^. Amongst the members of the DYRK family, DYRK4 has been classified as a strong HSP90 client, while DYRK1A, DYRK1B and DYRK2 are weak clients^10, 15^.

The recent discovery that rare heterozygous mutations of *DYRK1B* completely co-segregate with a familial form of metabolic syndrome raises the question how these mutations alter the properties of DYRK1B to give rise to this complex phenotype. The present study aims to elucidate the effects of the H90P and R102C mutations on a molecular and cellular level. Our results suggest that both mutations compromise the maturation by tyrosine autophosphorylation of DYRK1B, which leads to conformational instability and increased misfolding of the mutant proteins.

## Results

### Structural model of DYRK1B

**Figure 1:**
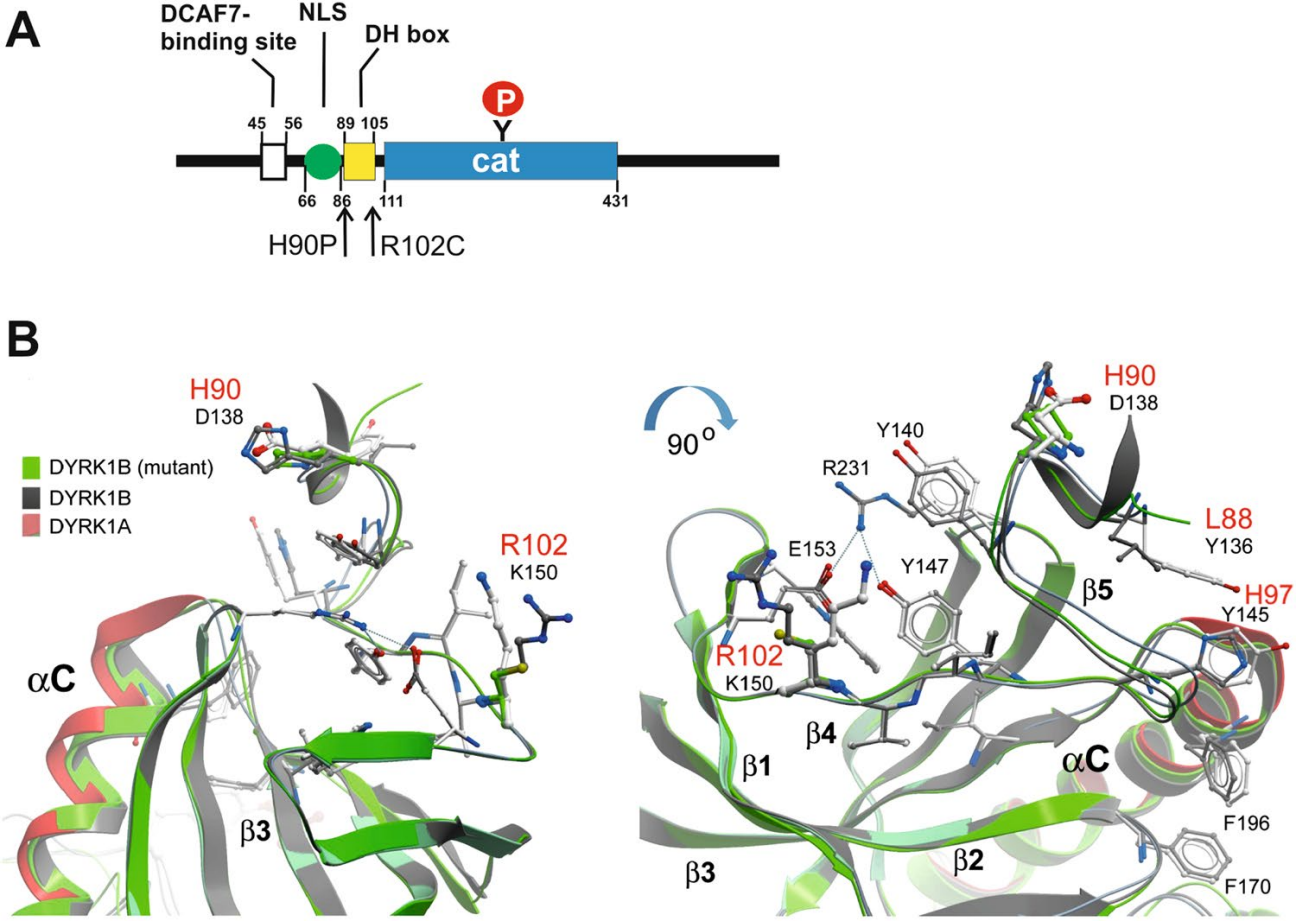
Location of the mutated amino acids in the N-terminal domain of DYRK1B. (**A**) Domain structure of DYRK1B. Sequence motifs in the N-terminal domain include a binding site for the adaptor protein DCAF7, a bipartite nuclear localization sequence (NLS) and the DH box. Autophosphorylation of Tyr273 in the catalytic domain (cat) is essential for the activation of DYRK1B. (**B**) Three-dimensional structural models of the DH box and N-lobe core of the catalytic domain. Shown is a superimposition of DYRK1A, DYRK1B and DYRK1B with substitutions of H90 by P and R102 by C. The ribbon diagrams have been colored as indicated. Two different views of the models are shown. DYRK1A residues are labelled in black while only these DYRK1B residues are highlighted in red that differ from DYRK1A. The main secondary structural elements are labeled (α**C**, β1-4).

Sequence analysis revealed that the residues affected by the pathogenic mutations belong to a conserved sequence motif designated “DYRK homology box” (DH box) in the N-terminal domain of DYRK1B (Fig. 1A)^1^. To date no experimental structure is available of DYRK1B. However, the high sequence conservation of the DYRK1A and DYRK1B isoforms allowed us to generate a homology model using available DYRK1A crystal structures^16, 17^. In the crystal structure of DYRK1A, the DH box participates in extensive interactions with the catalytic domain^17^. Comparisons of the generated model and the DYRK1A structure revealed that many of the DH box interactions are conserved between DYRK1A and DYRK1B, including the hydrogen bond network involving the central Y147, E153 and R231 (Y99, E105, R183 in DYRK1B) (Fig. 1B). However, a hydrophobic cluster (Y136, Y145) that fixes the N-terminal end of the αC helix in the active kinase conformation of DYRK1A is probably less efficiently stabilizing DYRK1B due to loss of hydrophobic and aromatic interactions (L88 and H97). Importantly, the mutated amino acids (H90 and R102) are exposed to the surface of the protein and do not make direct contacts with the catalytic domain. The missense mutations did not result in obvious structural perturbations of the active conformation of DYRK1B. It appears possible that the mutation of H90 to P may indirectly affect the backbone conformation of L88 and thereby indirectly disturb the positioning of the αC helix. It should be noted that our model does not include sequences N-terminal of the DH box and does not allow definitive conclusions regarding the effects of the missense mutations on the function of the kinase.

### *In vitro*-kinase activity of the pathogenic DYRK1B point mutants

**Figure 2:**
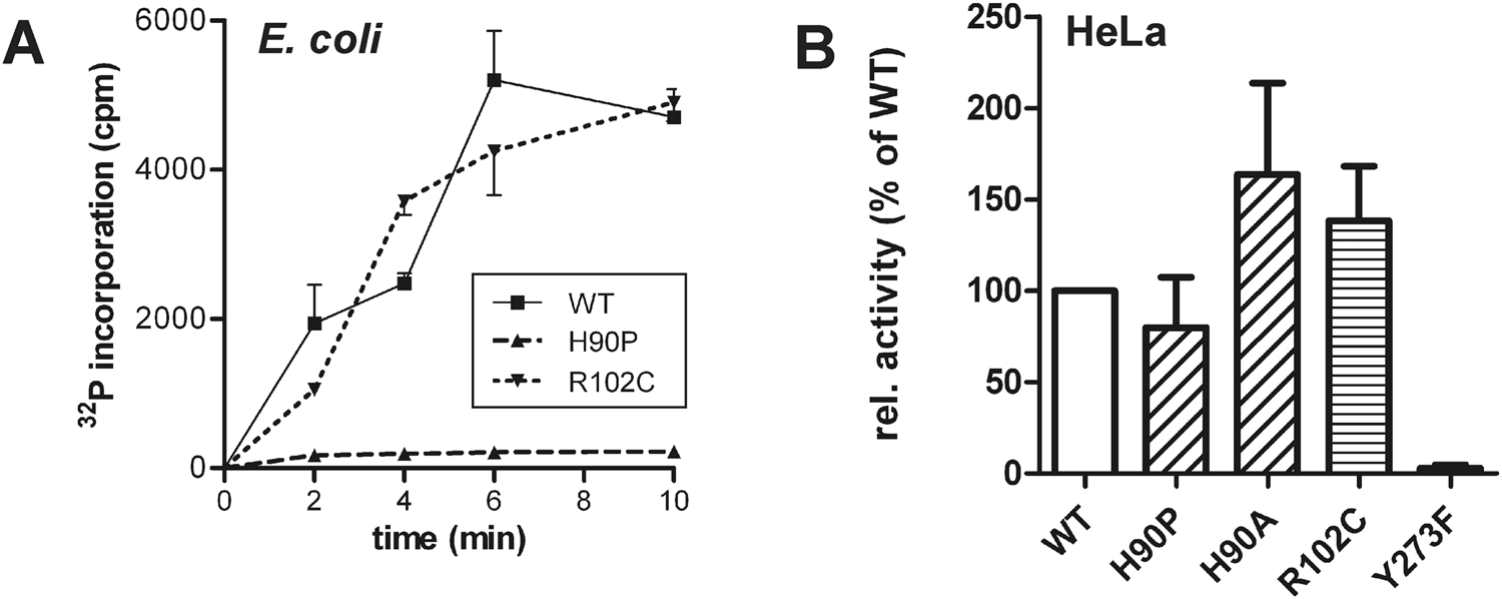
*In vitro* catalytic activities of wild type and mutant DYRK1B. (**A**) Wild type (WT) and mutant GST-DYRK1B fusion proteins were expressed in *E. coli*. Kinase activities were assayed by measuring the incorporation of [γ-^32^P] from radiolabeled ATP into the peptide substrate DYRKtide. The amounts of the recombinant kinases in the assay were adjusted according to their GST immunoreactivity on Western blots (bottom panel of Fig. 3A). The graph shows means and SD of triplicate measurement and is representative of three experiments. (**B**) HeLa cells transiently expressing wild type (WT) or mutant GFP-DYRK1B were extracted using a non-denaturing lysis buffer with the non-ionic detergent Igepal CA-630. GFP immunoprecipitates were subjected to radiometric kinase assays with the peptide substrate DYRKtide. *In vitro* kinase activities were normalized to the amount of the respective DYRK1B protein as quantified by immunoblot analysis. Columns show the results of 5 independent experiments (means and SD).

GST-DYRK1B fusion proteins were expressed in *E. coli* to investigate the effect of the mutations on the catalytic activity of the kinase. Radiometric *in vitro* kinase assays with the peptide substrate, DYRKtide, revealed DYRK1B-H90P as a catalytically inactive mutant, whereas the R102C mutation had no marked effect on kinase activity (Fig. 2A). GST-DYRK1B-H90P was also inactive in assays with two different substrate proteins (Suppl. Fig. S1).

We also conducted kinase assays with GFP-DYRK1B fusion proteins after immunoprecipitation from HeLa cells. The DYRK1B-H90A mutant was included to assess whether the loss of the histidine residue or rather its substitution by proline affect the kinase. DYRK1B-Y273F carries a mutation of the autoactivation site and served as a control with strongly reduced activity (~2% of wild type DYRK1B, Fig. 2B). Unlike the bacterial GST fusion protein, GFP-DYRK1B-H90P was hardly affected by the mutation, while H90A and R102C even appeared to be slightly more active. Taken together, these results suggest that the H90P mutation can impair the catalytic function of DYRK1B but that this negative effect is suppressed when the kinase is expressed in HeLa cells.

### Tyrosine autophosphorylation of the DYRK1B mutants

**Figure 3:**
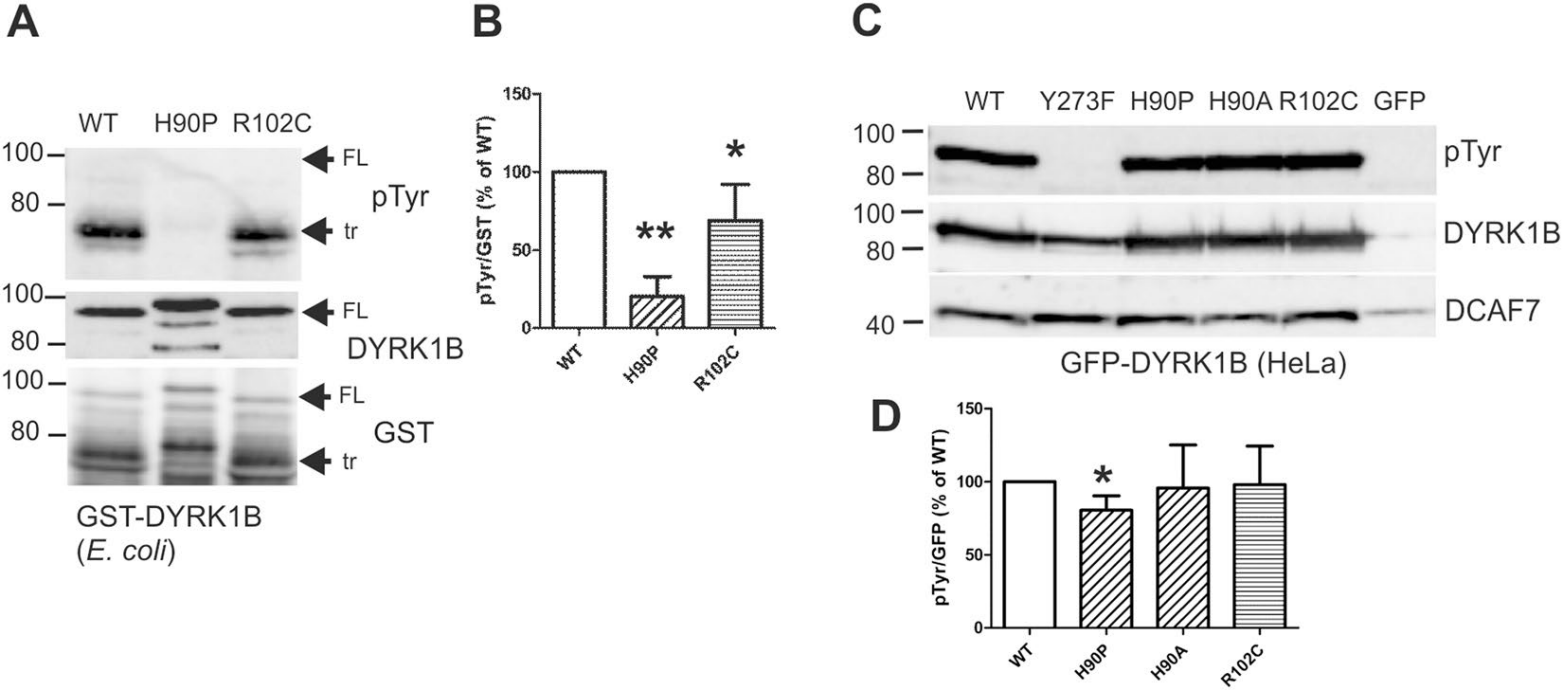
Tyrosine autophosphorylation of wild type and mutant DYRK1B. (**A**,**B**) Wild type and mutant GST-DYRK1B fusion proteins were purified from *E. coli* after overnight expression at 23 °C. These constructs resulted in C-terminally truncated (tr) protein and the full length (FL) protein. (**A**) Tyrosine-autophosphorylation was determined by immunoblot analysis with a phosphotyrosine-specific antibody (pTyr). (**B**) For quantification, pTyr band intensities were normalized for GST immunoreactivity. The column diagram shows the results (means and SD) of 5 independent experiments. (**C**,**D**) HeLa cells expressing wild type and mutant GFP-DYRK1B constructs were extracted using a non-denaturing lysis buffer with Igepal CA-630. GFP fusion proteins were immunoprecipitated and analysed by immunoblotting. The same blots were used to detect co-precipitated DCAF7. For quantification, pTyr band intensities were normalized for GFP immunoreactivity. Panel C shows the Western blots of a representative experiment and the column diagram (**D**) illustrates the results (means + SD) of 5 independent experiment (n = 3 for H90A). Significant differences between WT and mutant DYRK1B are marked (One sample t-test, *p < 0.05, **p < 0.01). Uncropped images of the Western blots are provided as supplementary material.

Next we investigated whether the pathogenic mutations affected tyrosine autophosphorylation of DYRK1B. When expressed in *E. coli*, GST-DYRK1B is isolated predominantly as a C-terminally truncated (tr) protein of about 70 kD and only a small amount of the full length (FL) protein with a calculated molecular mass of 95 kD (see the GST panel in Fig. 3A). GST-DYRK1B contains phosphotyrosine solely due to autophosphorylation, because *E. coli* is devoid of endogenous protein tyrosine kinase activity. Tyrosine autophosphorylation of GST-DYRK1B-H90P and GST-DYRK1B-R102C was reduced by 80% and 31%, respectively, as compared to the wild type kinase (Fig. 3A). GST-DYRK1B-H90P was also distinguished by its slower electrophoretic migration in SDS-PAGE. This band shift does not result from hyperphosphorylation, since it was resistant to phosphatase treatment (Fig. S2A). Reduced phosphotyrosine content and altered electrophoretic mobility was also observed for GST-DYRK1B-H90A (Suppl. Fig. S2B). This indicates that the band shift is due to the loss of the histidine residue rather than its substitution by the imino acid proline (which breaks α helix or β sheet structures).

We then determined the phosphotyrosine content of GFP-DYRK1B fusion constructs that were immunoprecipitated from HeLa cells. No phosphotyrosine was detected in DYRK1B-Y273F (Fig. 3C), confirming that tyrosine phosphorylation of DYRK1B is limited to the activation loop phosphorylation. GFP-DYRK1B-H90P showed a small reduction (20%) of the pTyr signal, whereas no significant effect of the R102C mutation was observed (Fig. 3C,D). We also examined whether the mutations might interfere with the interaction of the adaptor protein DCAF7 to its binding site in the vicinity of the DH box (Fig. 1A). Endogenous DCAF7 was co-immunoprecipitated equally well with wild type GFP-DYRK1B and all point mutants (Fig. 3C), indicating that DCAF7 binding was not altered in the mutant proteins.

### Ganetespib inhibits tyrosine autophosphorylation of DYRK1B

**Figure 4:**
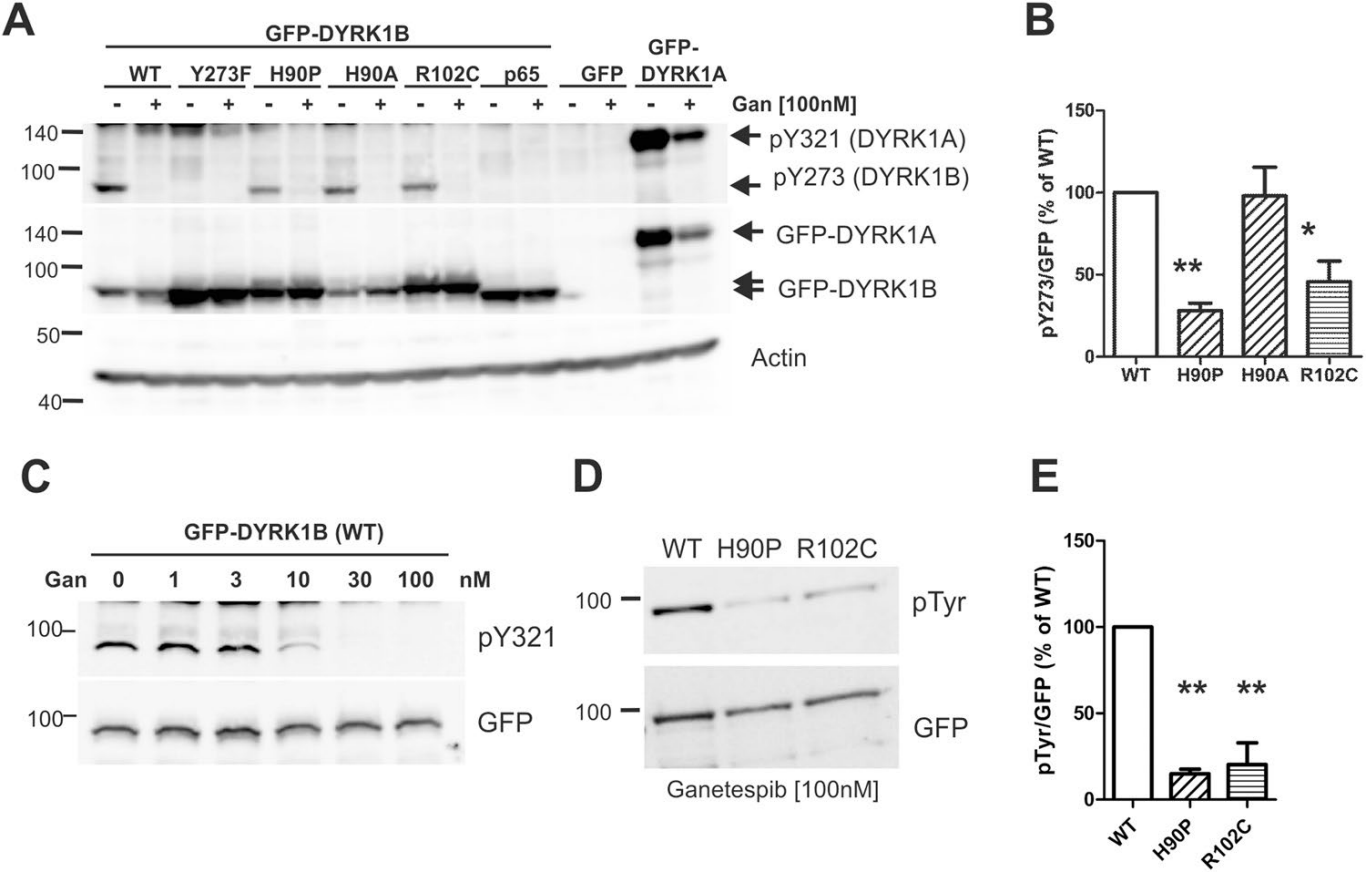
Effect of ganetespib on wild type and mutant DYRK1B. (**A**,**B**,**C**) Analysis of total cellular lysates. GFP-DYRK1B constructs were transiently expressed in HeLa cells as indicated. Cells were treated with ganetespib (100 nM) for 24 h before denaturing SDS lysis. Total cellular lysates were analysed for the phosphorylation of the activation loop tyrosine (Y273 in DYRK1B, Y321 in DYRK1A) using an antibody originally designed against the activation loop tyrosine in HIPK2. Actin was detected to control for equal loading. Panel A shows a the Western blots of a representative experiment, and panel B presents the quantification of the pY273 signal in the untreated cells relative to the GFP signal (means and SD; n = 3). In panel C, HeLa cells expressing wild type GFP-DYRK1B were treated with increasing concentrations of ganetespib for 24 h. (**D**,**E**) Analysis of immunoprecipitates HeLa cells were treated with ganetespib for 16 h and extracted using the non-denaturing lysis buffer with Igepal CA-630. GFP-DYRK1B constructs were immunoprecipitated and subjected to immunoblot analysis (**D**). Panel **E** illustrates the quantitative results of 3 independent experiments (means and SD). Significant differences between WT and mutant DYRK1B are marked (One sample t-test, *p < 0.05, **p < 0.01).

The differential effects of the H90P mutation in the bacterial and the mammalian expression system led us to hypothesize that the activity of chaperones may compensate the effect of the H90P mutation in HeLa cells. Thus, we investigated the effect of the HSP90 inhibitor, ganetespib, on tyrosine autophosphorylation of wild type and mutant GFP-DYRK1B. Inhibition of HSP90 can lead to either aggregation or degradation of client kinases^10^. In this experiment, total cell lysates were prepared under denaturing conditions, because we aimed to account for both soluble GFP-DYRK1B and possible aggregates. As a parameter for the functionality of catalytically active DYRK1B molecules, we determined the autophosphorylation of Tyr273. To this end, we took advantage of a commercial antibody that was developed as an activation state-specific antibody for HIPK2 (phospho-HIPK2-pY361). This antibody cross-reacts with the phosphotyrosines in the activation loop of DYRK1A (pY321) and DYRK1B (pY273), which are structurally related with HIPK2^18^. As a catalytically inactive control, we included DYRK1B-p65, a splicing variant that carries a deletion of 40 amino acids in the catalytic domain^17^. DYRK1A served as a control protein whose expression level is known to decrease in response to ganetespib treatment^15^. Consistent with this previous observation, ganetespib reduced DYRK1A levels without affecting its relative phosphotyrosine content (Fig. 4A). In contrast, ganetespib treatment did not alter the cellular amount of DYRK1B but largely eliminated tyrosine autophosphorylation of wild type and mutant DYRK1B. Ganetespib inhibited the autophosphorylation of wild type DYRK1B with a half-maximal effect at about 10 nM (Fig. 4C). Surprisingly, the normalized tyrosine phosphorylation of total cellular DYRK1B-H90P and DYRK1B-R102C in the untreated cells was lower than that of wild type DYRK1B (Fig. 4B). This result is in contrast to the above immunoprecipitation experiment (Fig. 3C) and is due to the different lysis methods used (see below).

Extended exposure of the blots revealed very weak phosphotyrosine signals in DYRK1B from ganetespib-treated cells (not shown). To improve the sensitivity of detection, we extracted the HeLa cells using a non-ionic detergent (Igepal CA-630) and immunoprecipitated the GFP-DYRK1B fusion proteins. As shown in Fig. 4D and E, treatment with ganetespib resulted in a reduced phosphotyrosine content of the mutant DYRK1B variants as compared to the wild type protein. This result suggests that the pathogenic mutations render DYRK1B more vulnerable to HSP90 inhibition.

### The pathogenic mutations increase aggregation of DYRK1B in HeLa cells

It is important to note that only soluble DYRK1B molecules were isolated by immunoprecipitation and analysed in Fig. 3C, whereas denaturing lysis by boiling SDS buffer also recovered protein aggregates (Fig. 4A). Thus, we hypothesized that the lower relative phosphotyrosine content of the H90P and R102C mutants in SDS lysates could be due to higher proportion unphosphorylated, insoluble DYRK1B.

**Figure 5:**
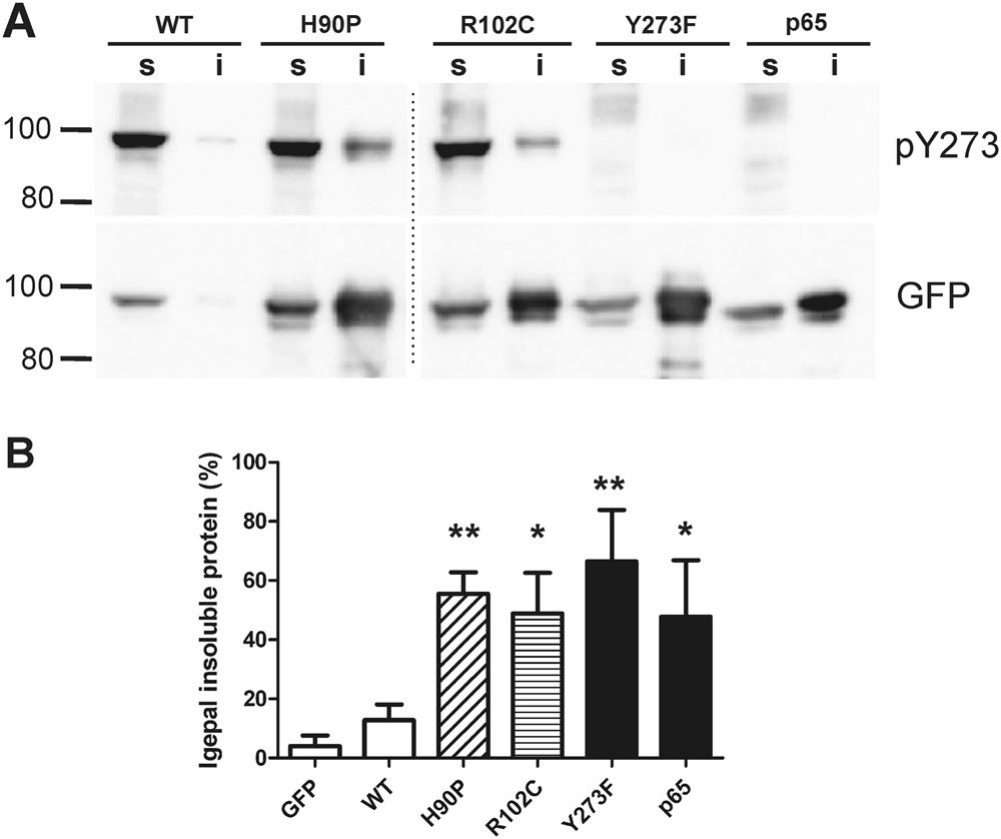
Differential aggregation of wild type and mutant DYRK1B variants. HeLa cells expressing wild type or mutant GFP-DYRK1B constructs were grown in 6-well plates and lysed using the non-denaturing immunoprecipation buffer with 0.5% Igepal CA-630. Lysates were centrifuged, and the distribution of the GFP proteins in the soluble and insoluble fraction was analysed by immunoblotting and by measuring GFP autofluorescence with the help of a microplate reader. (**A**) Western blot analysis. The gel was loaded with aliquots of the supernatants (24% of the total sample) and the pellets (50%). The dotted line indicates where irrelevant lanes were deleted from the final image. (**B**) Evaluation of GFP fluorescence measurements. Significant differences between WT and mutant DYRK1B are marked (two-tailed t-test, *p < 0.05, **p < 0.01) (means and SD, n = 4).

To test this hypothesis, we analysed the distribution of the GFP-DYRK1B constructs between the detergent-soluble fraction and the insoluble fraction. As calculated from the GFP fluorescence in the pellet and in the supernatant, about 10% of wild type GFP-DYRK1B was found in insoluble fraction. In contrast, less than 50% of GFP-DYRK1B-H90P and R102C was extracted with the non-ionic detergent Igepal CA-630 (Fig. 5). The same difference was observed at lower expression levels of DYRK1B-H90P and R102C, which excludes the possibility that excessive overexpression accounted for the aggregation of the proteins (Fig. S3B). These results suggest that the pathogenic mutations induce misfolding and aggregation of the mutant DYRK1B proteins when expressed in mammalian cells. A catalytically inactive point mutant (DYRK1B-D239N) was also predominately detected in the insoluble fraction (Fig. S3A), similar to the autophosphorylation incompetent variant, DYRK1B-Y273F (Fig. 5). Of note, tyrosine phosphorylation of insoluble DYRK1B-H90P and DYRK1B-R102C was much lower than that of the soluble molecules (Fig. 5A).

**Figure 6:**
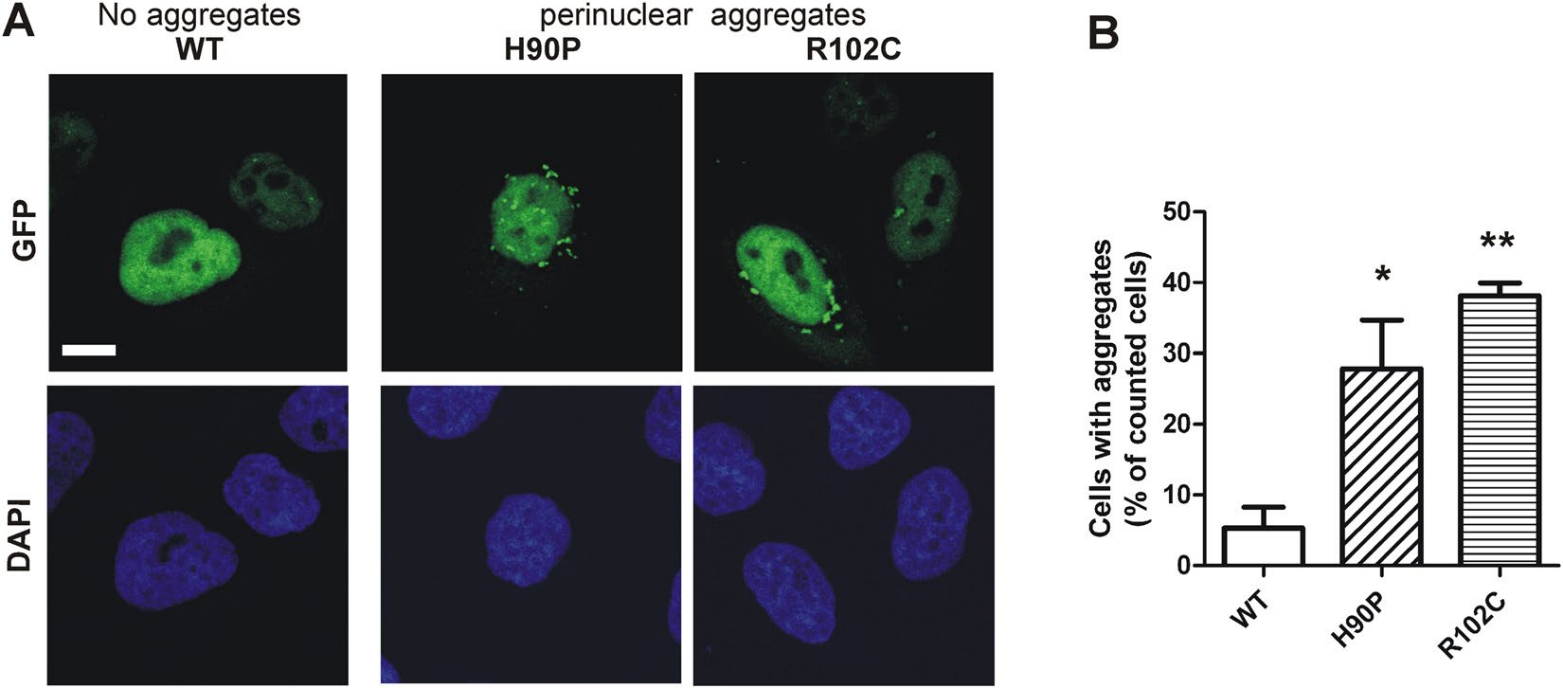
Detection of GFP-DYRK1B aggregates by fluorescence microscopy. GFP fluorescence was observed by confocal laser scanning microscopy two days after HeLa cells were transfected with expression plasmids for wild type or mutant GFP-DYRK1B. Panel **A** shows representative images of cells with and without aggregates. The percentage of cells with visible aggregates was determined in 3 independent experiments (panel **B**, means and SD). Statistical significance of the differences between WT and mutant DYRK1B was tested using paired Student’s t-test (*p < 0.05, **p < 0.01). Scale bar, 10 µm; DAPI, staining of nuclear DNA with 4′,6-diamidino-2-phenylindol.

Next we asked whether the presence of detergent-insoluble GFP-DYRK1B aggregates corresponded to differences in the subcellular localization of wild type and mutant DYRK1B. In agreement with previous observations^19^, HeLa cells expressing wild type GFP-DYRK1B showed even GFP fluorescence predominantly in the nucleus (Fig. 6A). Strikingly, punctate GFP fluorescence was observed in the vicinity of the nucleus of many cells that expressed the mutant GFP-DYRK1B constructs (H90P, R102C) (Fig. 6A). The higher propensity of mutant DYRK1B to form aggregates (Fig. 6B) suggests that these structures represent detergent-insoluble GFP-DYRK1B protein. Inhibition of proteasome activity by MG132 increased aggregate formation of wild type as well as mutant DYRK1B forms (Fig. S4). This result suggests that misfolded DYRK1B is partially degraded by the ubiquitin proteasome system and forms aggregates once the capacity of the proteasomal disposal pathway is exceeded.

### Effect of ganetespib on DYRK1B aggregation

**Figure 7:**
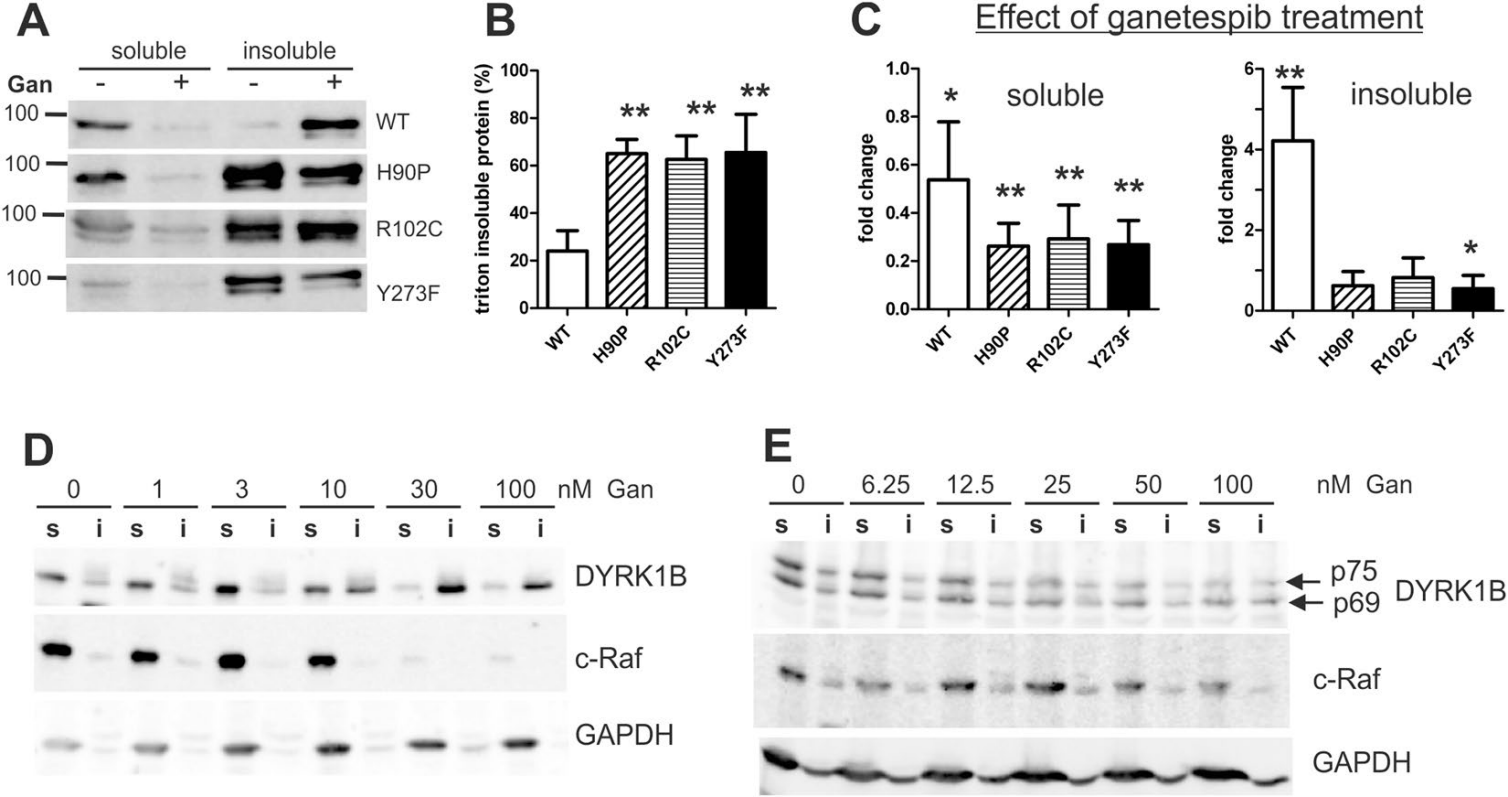
Effect of ganetespib on the aggregation of DYRK1B. (**A**–**C**) Effect on wild type and mutant GFP-DYRK1B. HeLa cells transiently expressing the indicated GFP-DYRK1B constructs were treated with 100 nM ganetespib for 24 h and then lysed with 0.1% Triton X-100, followed by separation of the soluble and insoluble fractions. Equivalent aliquots of the samples were subjected to Western blot analysis with a DYRK1B specific antiserum. Panel A shows the Western blots of a representative experiment. Blots from n = 5 independent experiments were densitometrically evaluated to determine the percentage of triton-insoluble protein in untreated cells (panel B) and the ganetespib-induced changes of GFP-DYRK1B levels (panel C). Significant differences between WT and mutant DYRK1B (panel B, two-tailed t-test) or treated and untreated cells (panel C, One sample t-test) are marked (*p < 0.05; **p < 0.01). (**D**,**E**) Concentration-response relationship of the ganetespib effect. Cells treated with increasing concentrations of ganetespib for 24 h before lysis with Igepal CA-630 containing buffer and fractionation. Western blots were developed with antibodies for DYRK1B, c-Raf as a known HSP90 client^42^, and GAPDH as a control for a HSP90-independent, stable protein. Relative to the soluble fraction, a 3-fold larger aliquot of the insoluble fraction was loaded on to the gel. In panel D, HeLa cells were transfected with untagged DYRK1B. Panel E shows untransfected RINm5F insulinoma cells. DYRK1B splicing variants were identified by co-migration with recombinant proteins (see uncropped blots). Blots are representative of 3 experiments.

Next we studied the effect of ganetespib on the aggregation of the DYRK1B variants. According to the protocol applied in the study of Taipale *et al*.^10^, cells were extracted with a buffer containing 0.1% Triton X-100 (Fig. 7A). Consistent with the results obtained with the Igepal-containing lysis buffer (Fig. 5), the pathogenic DYRK1B variants were predominantly detected in the detergent-insoluble fraction of untreated cells (Fig. 7B). Treatment with ganetespib reduced the amount of soluble protein for all constructs and resulted in the accumulation of wild type GFP-DYRK1B in the insoluble fraction (Fig. 7C). The distribution of the mutant DYRK1B variants was not significantly altered by ganetespib, possibly because the major part of these proteins was already in the insoluble fraction of untreated cells. Similar concentrations of ganetespib were required to induce the aggregation of DYRK1B and the degradation of the well-established HSP90 client, c-Raf (Fig. 7D). This experiment was performed with untagged DYRK1B, excluding the possibility that the conformational instability was due to the fusion with GFP. Finally, we studied the effect of HSP90 inhibition on endogenous DYRK1B in the RINm5F insulinoma cell line. In addition to the canonical p69 splicing variant that was used in the overexpression experiments, RINm5F cells express a longer splicing variant of DYRK1B (p75) with an extended N-terminal domain^17^. Treatment with ganetespib resulted in a dose-dependent reduction of DYRK1B and c-Raf levels in the soluble fraction (Fig. 7E). As compared with HeLa cells, RINm5F cells were relatively resistant to the effect of ganetespib. A recent survey revealed cell type-specific differences in the response to HSP90 inhibition for the majority of protein kinases that were identified as HSP90 clients^20^.

### The pathogenic mutations enhance the interaction of DYRK1B with CDC37

**Figure 8:**
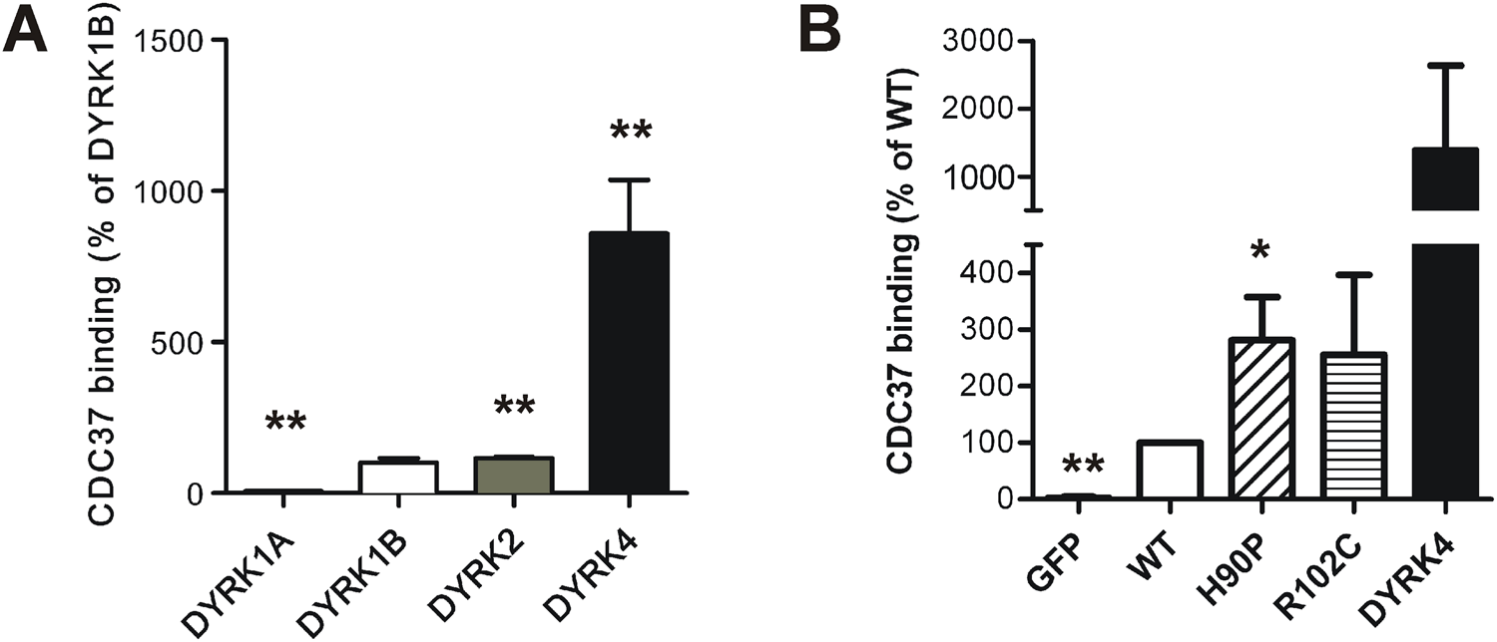
Interaction of DYRK family kinases and DYRK1B mutants with CDC37. (**A**) 293T cells stably expressing CDC37-nanoKAZ were transiently transfected with expression vectors for 3xFLAG-DYRK family kinases. Binding of CDC37-nanoKAZ to the kinases was determined from luminescence intensities relative to DYRK1B (means and SD, n = 8). (**B**) Wild type and mutant DYRK1B transfected in HeLa cells stably expressing CDC37-nanoKAZ. Association of CDC37-nanoKAZ with the immunoprecipitated GFP-DYRK1B constructs (WT, H90P, R102C) or GFP-DYRK4 is shown relative to wild type GFP-DYRK1B (means and SD, n = 4). Significant differences between wild type DYRK1B and other constructs are marked (two-tailed t-test, *p < 0.05, **p < 0.01).

The adaptor protein CDC37 mediates the interaction of HSP90 with protein kinases. Interestingly, binding of CDC37 to DYRK1A has been shown to be increased by point mutations that affect the autophosphorylation of DYRK1A. To analyze the interaction of DYRK kinases with CDC37, we used a fusion protein of CDC37 with a codon-optimized catalytic subunit of *Oplophorus* luciferase called nanoKAZ^15^. HEK293T cells stably expressing CDC37-nanoKAZ were transiently transfected with expression vectors for FLAG-tagged DYRK1A, DYRK1B, DYRK2 and DYRK4. Binding of CDC37-nanoKAZ to the kinases was determined using a microplate-based assay^15^. Among these kinases, DYRK4 showed the highest binding of CDC37-nanoKAZ (Fig. 8A), consistent with its classification as a strong HSP90 client^10^. DYRK1B and DYRK2 interacted more strongly with CDC37 than DYRK1A but not to the extent of DYRK4.

Next we asked whether the pathogenic mutation might impair maturation of DYRK1B by interfering with CDC37 binding. We used HeLa cell stably expressing CDC37-nanoKAZ to compare the interaction of CDC37 with wild type and mutant GFP-DYRK1B constructs. GFP served as a background control and DYRK4 was included as a strong client of the CDC37/HSP90 system. The GFP fusion proteins were isolated by immunoprecipitation, and interaction of CDC37-nanoKAZ with the kinase constructs was determined by luciferase assay and normalized to the amounts of the immunoprecipitated GFP fusion protein. As shown in Fig. 8B, both DYRK1B-H90P and DYRK1B-R102C interacted more strongly with CDC37 than wild type DYRK1B.

## Discussion

Kinase gene mutations affecting amino acids in the catalytic domain often alter protein function by reducing or enhancing kinase activity^21^. In contrast, the missense mutations in AOMS3 affect amino acids located outside the catalytic domain of DYRK1B, and it was one of the key questions whether these variants exert their pathogenicity via an altered kinase activity. Here we found that the mutant proteins exhibited normal catalytic activity when they were immunoprecipitated from the detergent-soluble fraction of HeLa cells. In contrast, DYRK1B-R102C was previously reported to exhibit reduced activity^6^. This discrepancy could be due to our shorter immunoprecipitation protocol, which may be less sensitive to differences in protein stability, or different procedures of cell lysis, which may result in the isolation of variable amounts of misfolded proteins. Nevertheless, our results suggest that H90 and R102 are not directly involved in the function of DYRK1B as a protein kinase. These residues are not evolutionarily conserved in vertebrate DYRK1B orthologs (Fig. S5), as it would be expected for amino acids that directly participate in enzyme function.

Apart from direct effects on protein function, protein misfolding events constitute the main other category of biochemical defects caused by missense mutations^22–24^. Protein folding problems can promote cell dysfunction in several ways, including enhanced degradation or accumulation of the misfolded protein, mislocalization in the cell, or structural alterations that establish novel toxic functions^23, 25^. Our experiments revealed that the majority of the mutant DYRK1B protein accumulated in the detergent-insoluble fraction of cultured cells, while wild type DYRK1B was efficiently solubilised with non-ionic detergents. Mutation of the tyrosine in the activation loop (Y273F) also resulted in a shift to the insoluble fraction, suggesting that the lack of tyrosine autophosphorylation promotes the aggregation of DYRK1B molecules. In agreement with this assumption, the mutant DYRK1B molecules in the insoluble fraction contained much less phosphotyrosine than the soluble DYRK1B protein. This explains why we found strongly reduced tyrosine phosphorylation of the mutant DYRK1B proteins in the total cell lysates (Fig. 4B), although the difference from wild type DYRK1B was marginal (H90P) or absent (R102C) when only the soluble protein fraction was analysed (Fig. 3D).

Kinases of the DYRK family attain their active conformation by one-time, irreversible tyrosine autophosphorylation immediately after translation^2, 3, 26^. Thus, the reduced phosphorylation of DYRK1B-H90P and R102C reflects the impaired maturation of the mutant proteins. In DYRK1A, the importance of the phosphorylated tyrosine for the conformation of the catalytic domain was clearly shown by the reduced thermal stability of the non-phosphorylatable mutant^16^. Therefore, it is plausible to assume that the defective maturation compromises the conformational stability of the DYRK1B mutants and renders the unphosphorylated proteins susceptible to misfolding and aggregation.

The co-chaperone CDC37 performs a quality control of protein kinases by scanning the conformational stability of client kinases^11, 27^. Therefore, CDC37 fused to luciferase can be used as a thermodynamic sensor for kinase structures^11, 15^. Here we show that the pathogenic DYRK1B variants interact more strongly with CDC37 than wild DYRK1B. This result matches well with previous studies of DYRK1A and GSK3, because point mutants of these kinases with reduced autophosphorylation also interact more strongly with HSP90/CDC37^15, 20, 28^. The enhanced binding of CDC37 to the autophosphorylation-incompetent mutants is in agreement with structural analyses of DYRK1A and GSK3, which have revealed that the phosphotyrosine in the activation loop stabilizes the catalytic domain^16, 29^. In striking contrast to DYRK1A and GSK3, the enhanced HSP90/CDC37 dependency of many oncogenic kinases such as v-Src or B-Raf^V600E^ results from activating rather than inactivating mutations^10, 13^.

The CDC37/HSP90 chaperone interacts with nascent and unfolded kinase chains and supports their folding^30, 31^. Here we show that the HSP90 inhibitor, ganetespib, interferes with tyrosine autophosphorylation of DYRK1B. This effect of ganetespib showed the same concentration-response characteristics (IC_50_ = 10–30 nM) as the inhibitory effect on the interaction of DYRK1A and CDC37^15^. Nevertheless, low levels of autophosphorylated wild type DYRK1B were detectable in ganetespib-treated cells (Fig. 4D) and in *E. coli* (Fig. 3A), which lacks the HSP90/CDC37 chaperone system. It should be noted that tyrosine autophosphorylation of bacterially expressed DYRK1B is rather inefficient if compared to DYRK1A^26^ and was only detectable when DYRK1B was expressed at room temperature. Interestingly, bacterially expressed DYRK1B-H90P was unphosphorylated and catalytically inactive, while this mutant was expressed as an active and autophosphorylated kinase in HeLa cells. It seems likely that the HSP90/CDC37 chaperone system can compensate to some degree for the deleterious effects of this mutation and enables autophosphorylation of DYRK1B-H90P in HeLa cells. The R102C mutation had only mild effects in *E. coli*, although both mutants behaved very similar in mammalian cells.

How can the missense mutations destabilize the catalytic domain although the affected amino acids are located in the N-terminal domain of DYRK1B? H90 and R102 belong to the DH box that is located immediately N-terminal of the catalytic domain^1^. Crystal structures of DYRK1A and DYRK2 revealed that the DH box stabilizes the active conformation of DYRKs by forming a large network of interactions with the catalytic domain^16^. Interestingly, deletion of the DH box in the *Drosophila* kinase dDYRK2 has been shown to reduce tyrosine-autophosphorylation and substrate phosphorylation activity^32^. Furthermore, single point mutations in the segment between the DH box and the catalytic domain eliminated tyrosine autophosphorylation of dDYRK2^33^. This result showed that sequences N-terminal of the catalytic domain can be critical for the maturation by tyrosine autophosphorylation and has led to the definition of the N-terminal autophosphorylation accessory (NAPA) regions, which also include a sequence motif further N-terminal. However, the whole N-terminal region is dispensable for the maturation of some other members of the DYRK family (DYRK1A, its *Drosophila* ortholog MNB, and DYRK2^26, 33, 34^.

**Figure 9:**
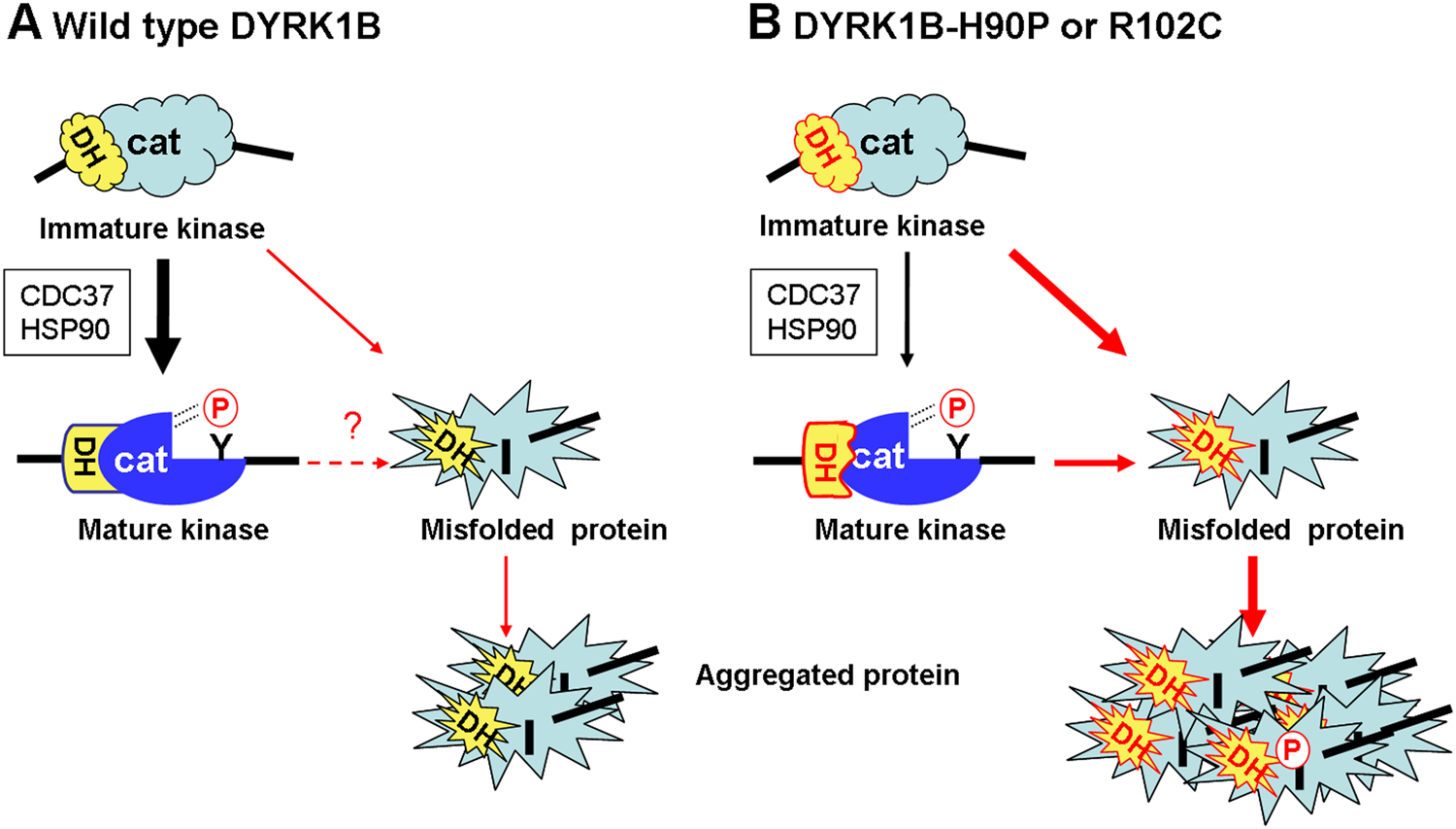
Proposed effect of the pathogenic mutations on the balance between DYRK1B maturation and misfolding. (**A**) DYRKs acquire the mature, active conformation of the catalytic domain by autophosphorylation (indicated by the circled P) of a tyrosine residue (**Y**) in the activation segment. In its folded state, the DH box forms interactions with the N-terminal lobe of the kinase domain^16^. The phosphorylated tyrosine (pY273 in DYRK1B) stabilizes the active conformation of the catalytic domain^16^. We propose that the unphosphorylated state is prone to misfolding and subsequent aggregate formation. The CDC37/HSP90 chaperone binds conformationally instable kinase domains and supports maturation. (**B**) We propose that the mutations in the DH box of DYRK1B perturb the maturation of the catalytic domain, which leads to enhanced misfolding and aggregation of unphosphorylated protein. The malfunction of the DH box also reduces the conformational stability of the mature protein, as evidenced by the presence of pY273 in the detergent-insoluble fraction and binding to CDC37.

Based on the present result, we propose that the mutations associated with AOMS3 impair the function of the DH box during maturation of DYRK1B. As illustrated in Fig. 9, newly translated DYRK molecules pass through a metastable folding intermediate that autophosphorylates on tyrosine to adopt the mature fully active conformation. Maturation of DYRK1B is largely dependent on HSP90. HSP90 inhibition thus leads to the misfolding of unphosphorylated kinase molecules. The DH box helps to adopt and maintain the active conformation. The pathogenic mutations in the DH box compromise maturation and thereby conformational stability. To some extent, the HSP90/CDC37 chaperone system compensates for the impaired function of the DH box and allows the formation of active, soluble DYRK1B molecules. However, even the mature, autophosphorylated mutant DYRK1B molecules appear to be destabilized, because they show enhanced interaction with CDC37. Furthermore, a small quantity of phosphorylated mutant proteins was detected in the insoluble fraction, suggesting that the DH box is still needed to maintain the active conformation once tyrosine autophosphorylation has taken place. This is reminiscent of mutant v-Src, which depends on constitutive HSP90 association, while c-Src requires HSP90 only at an early stage for maturation^35^.

In conclusion, we provide evidence that the missense mutations render DYRK1B susceptible to misfolding and degradation or aggregation and thus confer a loss of biochemical function rather than a gain of function. Although loss-of-function mutations are typically inherited in a recessive fashion, it is possible that mutant DYRK1B aggregation constitutes a gain of toxic function, similar to mutant tau or synuclein in familial forms of neurodegenerative diseases. Alternatively, a strong gene dosage effect of *DYRK1B* may account for the dominant inheritance of AOMS3 just as haploinsufficiency *of DYRK1A* causes a syndrome of autosomal dominant mental retardation (MRD7)^36^. This possibility is supported by the fact that the large-scale analysis of human genetic variation by the Exome Aggregation Consortium (ExAC) has identified *DYRK1B* as a loss-of-function intolerant gene, as expected for haploinsufficient disease genes^37^. Further research will be necessary to bridge the gap between the molecular effects of the mutations and their involvement in the etiology of the disease.

## Materials and Methods

### Antibodies

The following antibodies were commercially purchased: rabbit polyclonal antibodies against phospho-HIPK2 (pTyr361) (Thermo Scientific, #PA5-13045) and c-Raf (Cell Signaling Technology, #9422), rabbit monoclonal antibodies against GAPDH (Cell Signaling Technology, #2118), smooth muscle actin and DCAF7 (Abcam, Cambridge, UK; ab32575 and ab138490), goat polyclonal antibody against GFP (Rockland Immunochemicals, Gilbertsville, PA, USA), mouse monoclonal antibody against phosphotyrosine (PY99, Santa Cruz Biotechnology, sc-7020). A custom-made rabbit polyclonal antiserum against the C-terminus of DYRK1B has been described before^28^.

### Plasmids

Vectors for bacterial expression of GST-DYRK1B and mammalian expression of untagged DYRK1B-p69, GFP-DYRK1B, GFP-DYRK1A, GFP-DYRK4 and CDC37-nanoKAZ have been described previously^15, 17, 19, 38, 39^. Point mutants were created with the help of the QuikChange Site-Directed Mutagenesis Kit (Stratagene, La Jolla, CA, USA) using the DYRK1B-p69 splicing variant as template^17^. Expression vectors for GST-E1A-X2 and GST-p27^Kip1^ were reported before^8, 40^.

### Bacterial expression

*E. coli* DH5α cells were grown to the early logarithmic phase (optical density at 600 nm of about 0.2) at 37 °C before expression was induced by addition of IPTG (final concentration 1 mM). Expression of GST-E1A-X2 and GST-p27^Kip1^ proceeded for 2 h at 37 °C before lysis. For expression of GST-DYRK1B, cultures were grown overnight at 23 °C. Cells were lysed on ice in PBS supplemented with 1 mM phenylmethylsulfonyl fluoride, 166 µg/mL lysozyme and 1% (v/v) Triton X-100 and GST fusion proteins were isolated by affinity adsorption to glutathione Sepharose (GE Healthcare, Freiburg, Germany). Bound proteins were eluted with glutathione elution buffer (50 mM Tris-Cl pH 8.0, 10 mM reduced glutathione) for use in kinase assays or with Laemmli’s sample buffer containing dithiothreitol (30 mg/mL) for successive Western blot analysis.

### Cell culture and transfection

HeLa cells were maintained in TumorPlus 263 medium (Capricorn Scientific GmbH, Ebersdorfergrund, Germany) or RPMI1640 supplemented 10% fetal calf serum at 37 °C and 5% CO_2_. Cells were transfected with plasmid DNA using Fugene HD (Promega, Mannheim, Germany). HeLa cells with stable expression of CDC37-nanoKAZ from the pCAGIPuro vector^15^ were selected with puromycin for 2 weeks. If indicated, cells were treated with either ganetespib (Selleck Chemicals, Houston, TX, USA) dissolved in DMSO. Rat RINm5F insulinoma cells were available in the lab from earlier projects and grown in RPMI 1640 medium as described^41^.

### Buffers used for cell lysis and fractionation

Denaturing cell lysis by hot SDS lysis buffer (20 mM Tris-Cl pH 7.5, 1% SDS; prewarmed at 95–100 °C) was applied to recover the total cellular proteins for Western blot analysis (Fig. 4A–C). Lysates were incubated for 5 min at 96 °C, sonicated, and cleared by centrifugation, although no visible pellet was detectable.

Non-denaturing lysis buffer with Igepal-CA630 (50 mM Tris-Cl pH 7.5, 150 mM NaCl, 15% glycerol, 2 mM EDTA, 0.5% Igepal-CA630, supplemented with 1 mM Na_3_VO_4_, 1 mM phenylmethylsulfonyl fluoride and 10 µg/ml each of aprotinin, pepstatin and leupeptin, precooled on ice) was used for immunoprecipitation experiments (Figs 2B, 3C+D, 4D+E) and for separating the soluble and insoluble fractions (Figs 5, 7D,E, Fig. S3). Plates were agitated on ice for 20 minutes before the lysates were transferred to test tubes, sonicated and centrifuged (5 min, 14.000 rpm, 4 °C).

Alternatively, a Triton-containing buffer (0.1% Triton X-100, 50 mM Tris-Cl pH 7.5, 150 mM NaCl, 1 mM EDTA) was used for lysis in the experiment shown in Fig. 7A–C, according to the conditions applied by Taipale *et al*.^10^.

### Immunoprecipitation

Cells were washed in ice-cold PBS and then lysed with non-denaturing lysis buffer containing 0.5% Igepal CA-630 (see above, 1 mL per 10-cm plate). Plates were agitated on ice for 20 minutes. Lysates were sonicated and cleared by centrifugation. The supernatants were then incubated with 10 μL of GFP-Trap_M (ChromoTek, Martinsried, Germany) in an end-over-end rotator for 1 h at 4 °C. The paramagnetic beads of GFP-Trap_M were washed twice with washing buffer (50 mM Tris-Cl pH 7.5, 150 mM NaCl, 2 mM EDTA, 0.1% Igepal CA-630) and collected using a magnetic rack. Bound proteins were eluted by adding 20 μL Laemmli sample buffer containing 6 μg/100 μL dithiothreitol at 95 °C for 5 minutes.

### *In vitro*-kinase assays

For radiometric assays, GST-DYRK1B was incubated with the peptide substrate DYRKtide (final concentration 100 µM) in kinase buffer (25 mM Hepes pH 7.4, 0.5 mM dithiothreitol, 5 mM MgCl_2_) in the presence of 100 µM [γ-^32^P]ATP (Hartmann Analytics, Braunschweig, Germany) at 30 °C. Phosphate incorporation was determined by the phosphocellulose method. For the non-radioactive assays with protein substrates, GST-DYRK1B was incubated with GST- p27^Kip1^ (100 ng/µL) or GST-E1A-X2 in the presence of 500 µM ATP at 30 °C for variable times as indicated in Figure S1. Kinase activity was monitored by immunoblot analysis using phosphospecific antibodies. The amount of GST-DYRK1B present in the assays was adjusted according to quantification by immunoblot analyses.

GFP-DYRK1B fusion proteins were immunoprecipitated from transiently transfected HeLa cells with the help of GFP-Trap_M as described above. Samples were assayed for kinase activity with 20 µM DYRKtide and 10 μM [γ-^32^P]ATP in a volume of 20 μL for 10 min at 30 °C. Incorporation of ^32^P into DYRKtide was determined in triplicate by the phosphocellulose method and the linearity of the reaction was verified by measuring the incorporation of ^32^P after 20 minutes. GFP was used as background control. The amounts of GFP-DYRK1B protein in the assays was measured by Western blot assays.

### Western blotting

Samples were separated by SDS-PAGE in 8% acrylamide gels and transferred to nitrocellulose membranes by tank blotting. Afterwards membranes were blocked in 3% BSA/TBST and incubated with primary antibodies at 4 °C overnight. After washing and incubation with HRP-coupled secondary antibodies for 1 h at room temperature, chemiluminescence signals were detected with a LAS-3000 CCD imaging system (Fujifilm). Densitometric signal quantification was performed with AIDA image analysis software (Raytest, Straubenhardt, Germany).

### Ganetespib treatment

HeLa cells were cultivated in six-well plates and transfected with the indicated GFP-DYRK1B fusion proteins, or the empty vector. Cells were treated with either ganetespib (Selleck Chemicals, Houston, TX) to a final concentration of 100 nM or vehicle (DMSO) 24 h after transfection. Cells were washed in ice-cold PBS and lysed in hot SDS lysis buffer (20 mM Tris-Cl pH 7.5, 1% SDS) 24 hours after treatment. Samples were heated to 100 °C for 5 minutes, sonicated and cleared by centrifugation before SDS-PAGE.

### Fractionation of soluble and insoluble proteins

For separation of soluble and insoluble protein, cells were extracted with the non-denaturing lysis buffer containing Igepal CA-630 or Triton X-100 as described above. Cells were agitated on ice for 20 min and sonicated before centrifugation (5 min, 14.000 rpm, 4 °C). Supernatants were collected as the soluble fraction, and the pellets were resuspended in 50 µL 1% SDS, 20 mM Tris pH 7.5 by vortexing and incubation for 10 min at room temperature and a second incubation step for 5 min at 35 °C. Supernatants and the resuspended pellets (insoluble fraction) were either subjected to fluorescence measurement in a Tecan GENios microplate reader (Fig. 5) or analysed by Western blotting. Fluorescence of supernatants and pellets from untransfected cells was subtracted as background before the percentage of insoluble autofluorescent protein was calculated. Aliquots of the samples were analysed by immunoblotting.

### Fluorescence microscopy

HeLa cells were seeded on coverslips in 12-well plates and transiently transfected with expression plasmids for GFP-DYRK1B (0.225 µg DNA per well). When indicated, cells were treated with 20 µM MG132 for 4 h before fixation. Two days after transfection, cells were fixed with 4% paraformaldehyde for 10 min, permeabilized with 0.1% Triton X-100 in PBS for 10 min, stained with 0.3 µg/mL DAPI in PBS for 10min, washed twice with PBS and mounted in ProLong Gold (Invitrogen). Confocal laser scanning microscopy was performed on a TCS SP5 AOBS system (Leica Microsystems) and images were acquired using an HCX PL APO 63 × /1.4NA oil-immersion objective and LAS AF software (Leica Microsystems). For quantitative evaluation, cells were classified into two types: cells without cytoplasmic aggregates, and cells with clear cytoplasmic aggregates. We evaluated the cell type and counted the number of both cell types in 40–60 GFP-positive cells for each sample.

### Assay of CDC37 binding

Interaction of the FLAG-tagged DYRK family kinases with CDC37 was measured in HEK293 cells stably expressing CDC37-nanoKAZ using the microplate-based assay as described previously^15^. In essence, cleared cell lysates were incubated in a 96-well plate coated with FLAG antibody at 4 °C for 3 h and binding of CDC37-nanoKAZ to the 3xFLAG-DYRK proteins was determined as luminescence intensities. Luminescence data were normalized to the amounts of 3xFLAG protein bound on the antibody-coated well as quantified with a horseradish peroxidase (HRP) conjugated antibody against FLAG.

HeLa cells stably expressing CDC37-nanoKAZ were used for the analysis of the GFP-DYRK1B mutants. Cells were grown in 6-well plates and lysed in 140 µL ice-cold HENG buffer (50 mM HEPES-KOH, pH 7.9, 150 mM NaCl, 20 mM Na_2_MoO_4_, 2 mM EDTA, 5% glycerol, 0.5% Triton X-100) supplemented with Na_3_VO_4_ and protease inhibitors. Half of the lysates was subjected to immunoprecipitation with 5 µL GFP-Trap_M (1 h at 4 °C in a total volume of 470 µL). The beads were washed three times with ice-cold HENG buffer before two 10%-aliquots of the resuspended beads were taken for luciferase assays with 1 µM *bis*-coelenterazine as substrate (coelenterazine 400a, Cayman Chemical, Ann Arbor, MI, USA). Luminescence was recorded for 12 s using a Lumat LB 9507 Luminometer (Berthold Technologies, Bad Wildbad, Germany). The remaining immunoprecipitate was analysed by immunoblotting with a GFP-specific antibody for normalization to the amount of the baits.

### Structural modeling

Structural modelling of DYRK1B and the double mutant H90P and R102C has been carried out using the Swiss modeller software selecting the structure of DYRK1A as a template (pdb-ID: 2WO6).

### Statistics

The One sample t-test was applied to test the hypothesis that the readout parameter was different between wild type DYRK1B and a particular DYRK1B mutant, or between ganetespib-treated and untreated cells (pairwise comparison).

## Electronic supplementary material

Supplementary Information
