# Supplementary material for: DYRK1B mutations associated with metabolic syndrome impair the chaperone-dependent maturation of the kinase domain
Source: Sci Rep. 2017 Jul 25;7:6420. doi: 10.1038/s41598-017-06874-w (PMC5526990; doi:10.1038/s41598-017-06874-w)

Supplementary Material

DYRK1B mutations associated with metabolic syndrome impair the chaperone-dependent maturation of the kinase domain

Samira Abu Jhaisha[1], Esti W. Widowati[1,2], Isao Kii[3], Rie Sonamoto[3], Stefan Knapp[4], Chrisovalantis Papadopoulos[1,5], Walter Becker[1*]

1 Institute of Pharmacology and Toxicology, RWTH Aachen University, Aachen, Germany
2 Chemistry Study Program, Faculty of Science and Technology, State Islamic University (UIN) Sunan Kalijaga, Yogyakarta, Indonesia
3 Pathophysiological and Health Science Team, Imaging Platform and Innovation Group, Division of Bio-Function Dynamics Imaging, RIKEN Center for Life Science Technologies, 6-7-3 Minatojima-minamimachi, Chuo-ku, Kobe 650-0047, Japan
4 Institute for Pharmaceutical Chemistry and Buchmann Institute for Molecular Life Sciences (BMLS), Johann Wolfgang Goethe University, Frankfurt am Main, 60438, Germany.
5 current address: Molecular Biology I, Center for Medical Biotechnology, University of Duisburg-Essen, Essen, Germany

Supplementary figures

Fig. S1: *In vitro*-phosphorylation of E1A and p27^{kip1} by wild type and mutant DYRK1B.

Fig. S2: Reduced electrophoretic mobility of GST-DYRK1B-H90P.

Fig. S3: Aggregation of wild type and mutant DYRK1B

Fig. S4: DYRK1B aggregates in HeLa cells after treatment with MG132

Fig. S5: Sequence alignment of the DH box from DYRK1B.

Uncropped Western blots

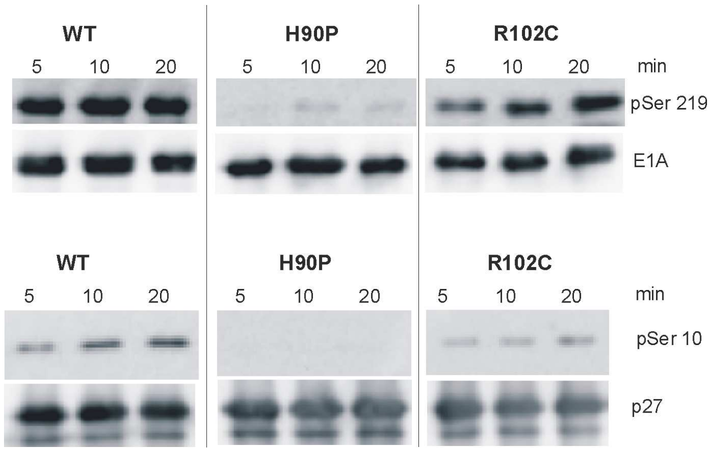

Fig. S1: ***In vitro*****-phosphorylation of E1A and p27**$^{\mathbf{kip1}}$ **by wild type and mutant DYRK1B.** Amount of wild type and mutant GST-DYRK1B were adjusted according to their immunoreactivity on Western blot. Kinase assays were performed at 30°C using GST-E1A and GST-p27 as substrates for the indicated times. Substrate phosphorylation was detected by Western blot analysis with antibodies directed against the DYRK target sites in the substrate proteins.

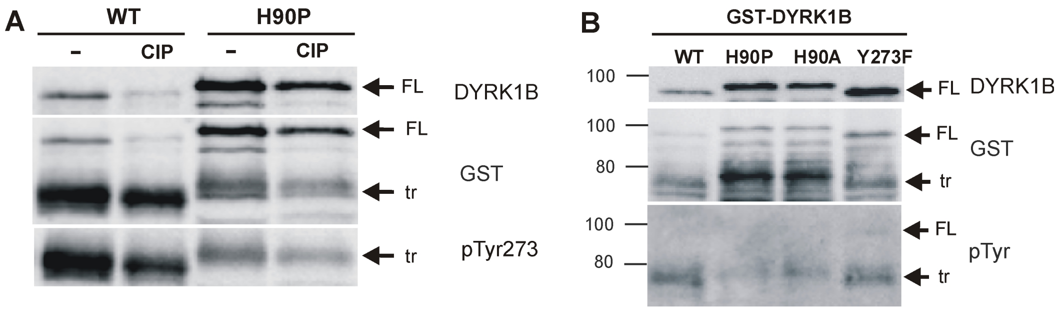

Fig. S2: Reduced electrophoretic mobility of GST-DYRK1B-H90P.

A) Treatment with calf alkaline phosphatase (CIP) does not alter migration of GST-DYRK1B-H90P.

B) GST-DYRK1B-H90A shows the same upshift and reduced phosphotyrosine content as GST-DYRK1B-H90P.

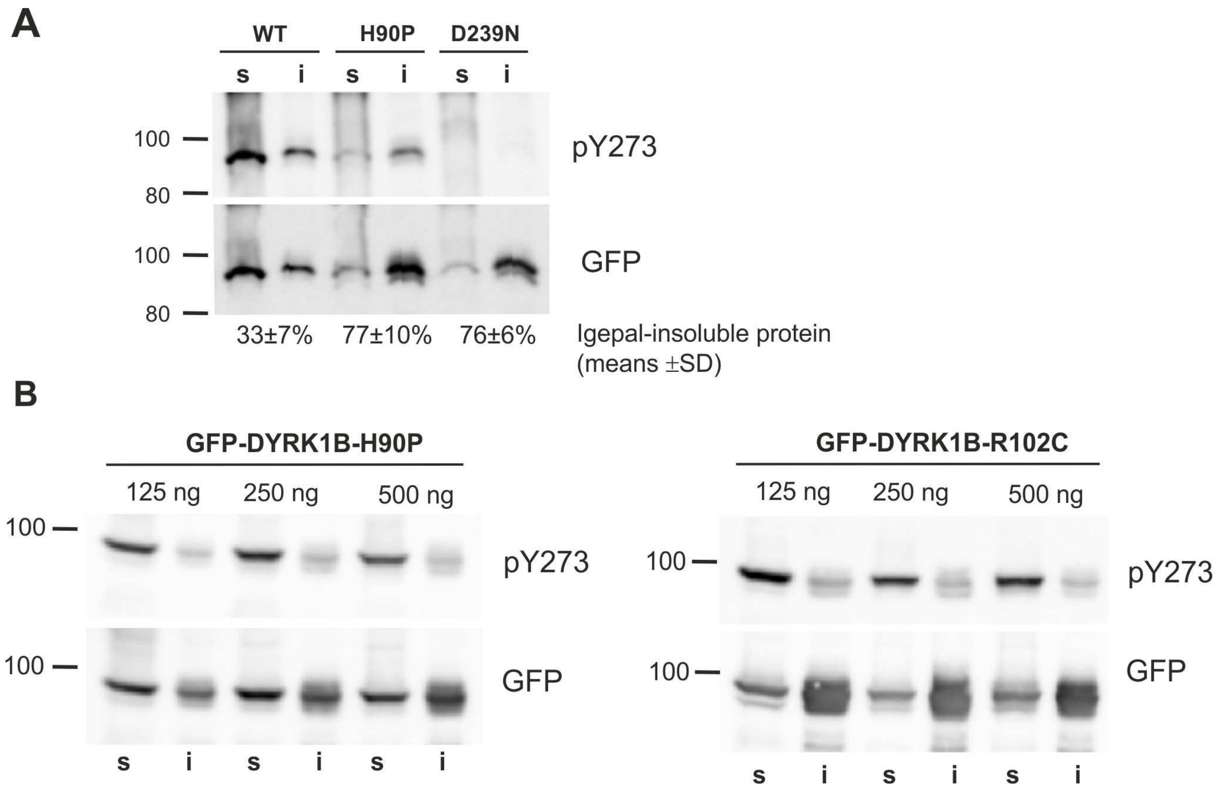

Fig. S3: Aggregation of wild type and mutant DYRK1B

HeLa cells were transfected with GFP-DYRK1B expression plasmids as indicated. Cell lysates were centrifuged to separate detergent-soluble (s) and insoluble (i) fractions as in the experiment shown in Fig. 5.

A) Aggregation of the catalytically inactive mutant DYRK1B-D239N. Results of the densitometric evaluation of 3 independent experiments are given below the blots.

B) Aggregation of DYRK1B-H90P and R102C at variable expression levels.

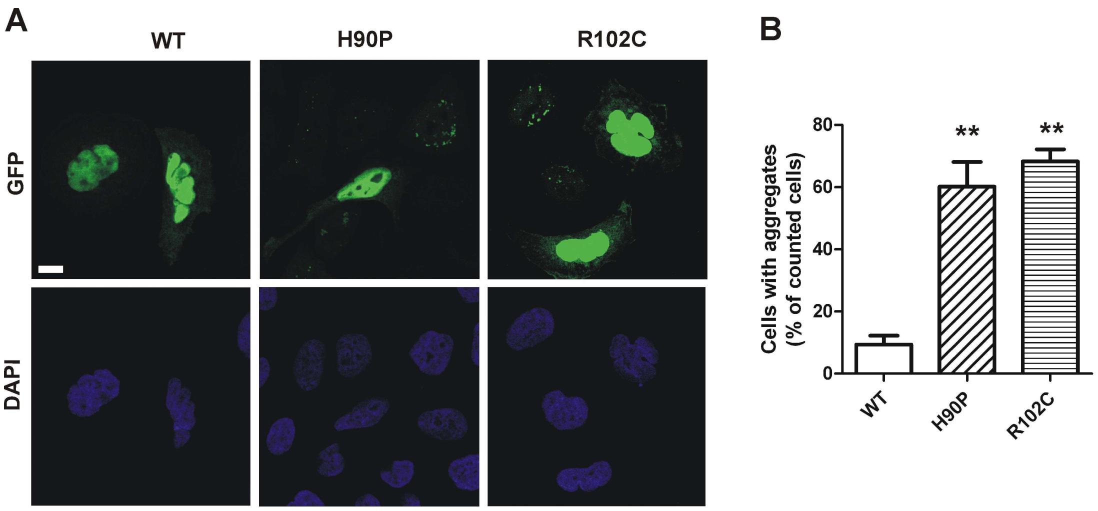

Fig. S4: DYRK1B aggregates in HeLa cells after treatment with MG132

GFP fluorescence was observed two days after transfection. HeLa cells were treated with 20 µM MG132 for 4 h before fixation. Panel A shows typical images of transfected cells without aggregates, which often showed distorted and intensely stained nuclei, and cells with punctate aggregates and weak or absent nuclear staining. The percentage of cells with visible aggregates was determined in 3 independent experiments (panel B, means and SD). Statistical significance of the differences between WT and mutant DYRK1B was tested using paired Student's t-test (* $p < 0.05$, **, $p < 0.01$).

Scale bar, 10 µm; DAPI, staining of nuclear DNA with 4′,6-diamidino-2-phenylindol.

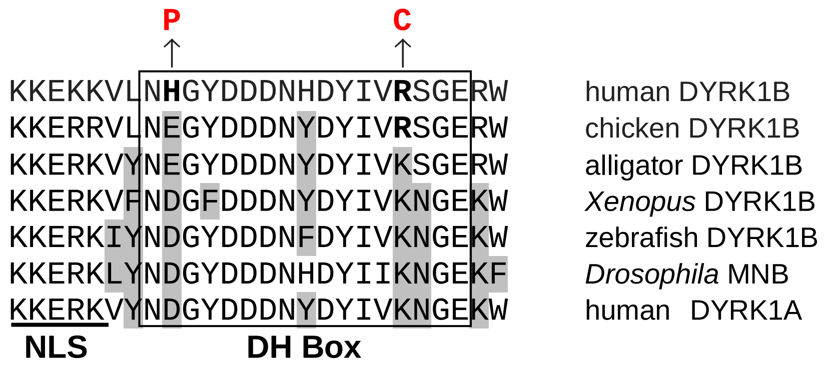

Fig. S5: Sequence alignment of the DH box from DYRK1B

The alignment illustrates that H90 and R102 are neither conserved in DYRK1B from other vertebrates nor in DYRK1A or the *Drosophila* DYRK1 ortholog, minibrain (MNB). Note however that both residues are conserved in all mammalian DYRK1B sequences. Amino acids deviating from the human DYRK1B are shaded.

Uncropped Western blots

Fig. 3A

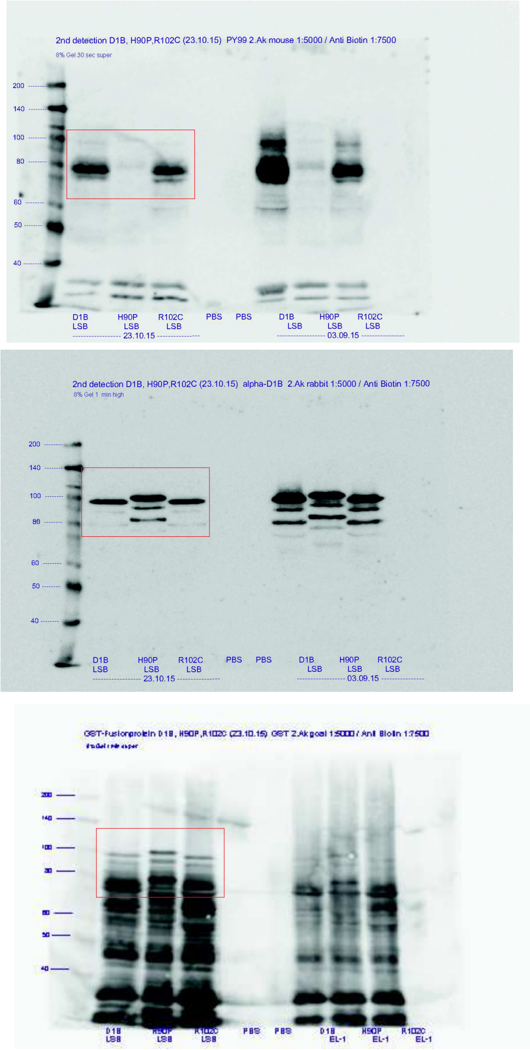

Fig. 3C

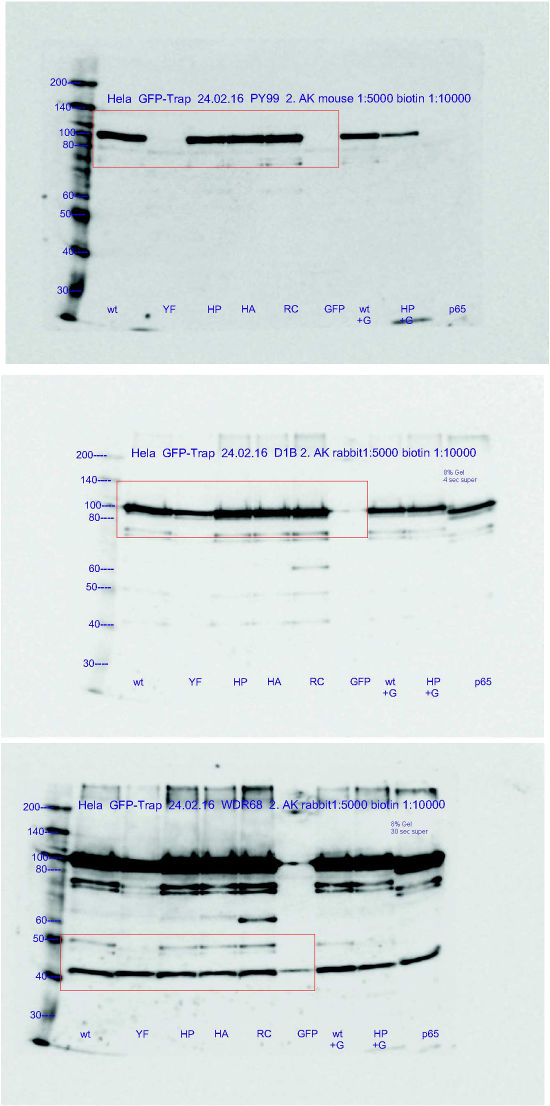

Fig. 4A

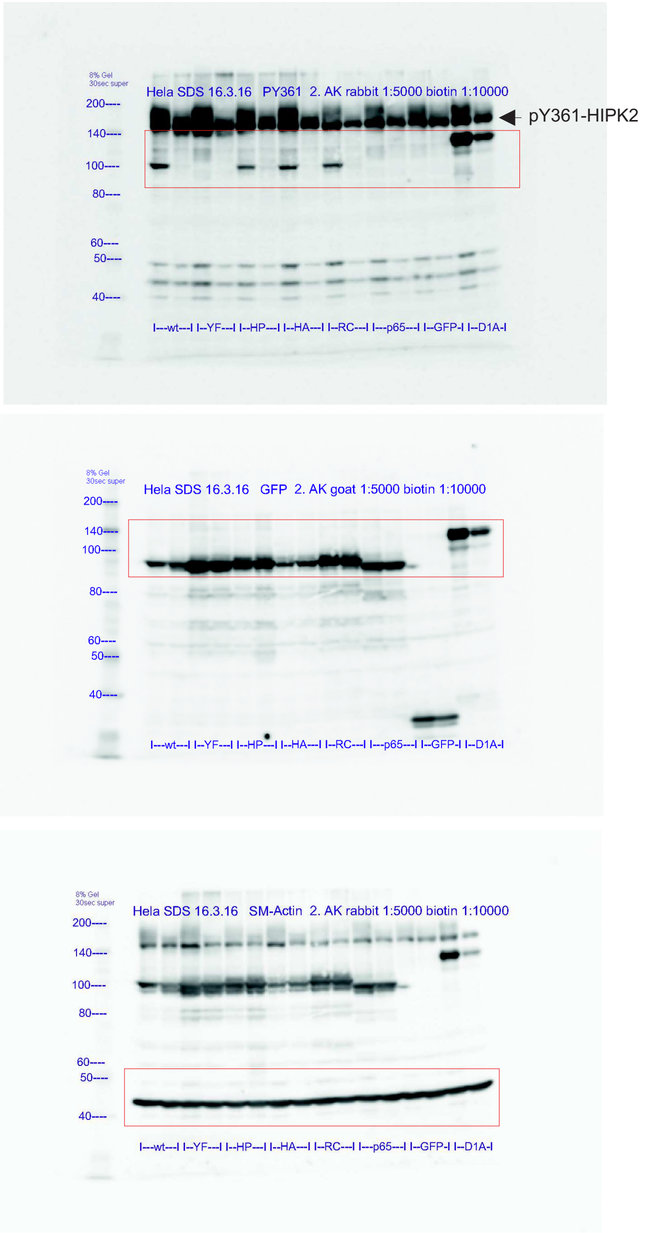

Fig. 4C

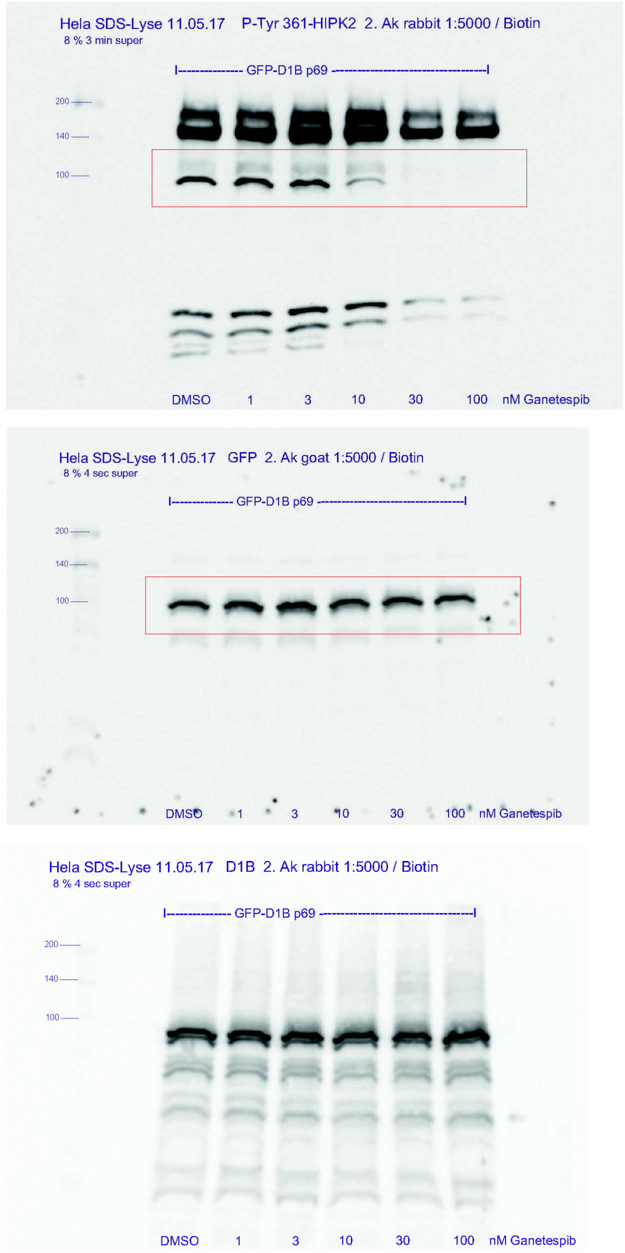

Fig. 4D

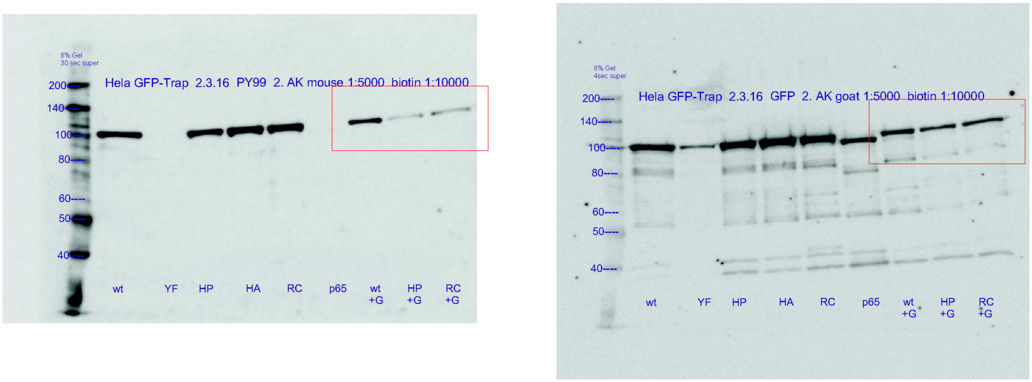

Fig. 5A

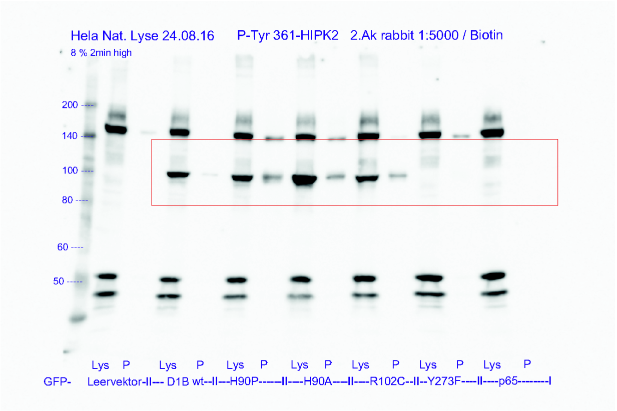
Hela Nat. Lyse 24.08.16
P-Tyr 361-HIPK2
2.Ak rabbit 1:5000 / Biotin
8 % 2min high
200
140
100
80
60
50
Lys P Lys P Lys P Lys P Lys P Lys P Lys P
GFP- Leervektor-II--- D1B wt--II---H90P------II----H90A----II----R102C--II--Y273F----II----p65--------I

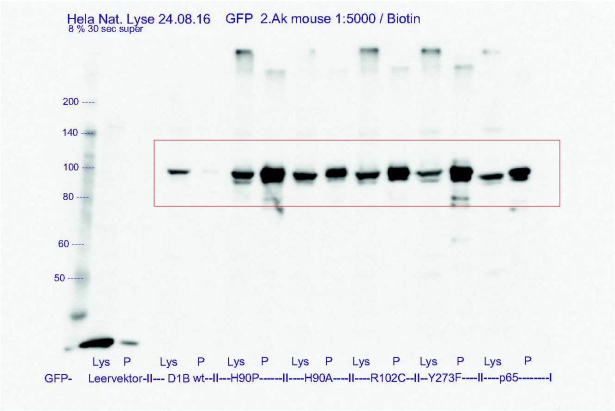
Hela Nat. Lyse 24.08.16
GFP 2.Ak mouse 1:5000 / Biotin
8 % 30 sec super
200
140
100
80
60
50
Lys P Lys P Lys P Lys P Lys P Lys P Lys P
GFP- Leervektor-II--- D1B wt--II---H90P------II----H90A----II----R102C--II--Y273F----II----p65--------I

Fig. 7A

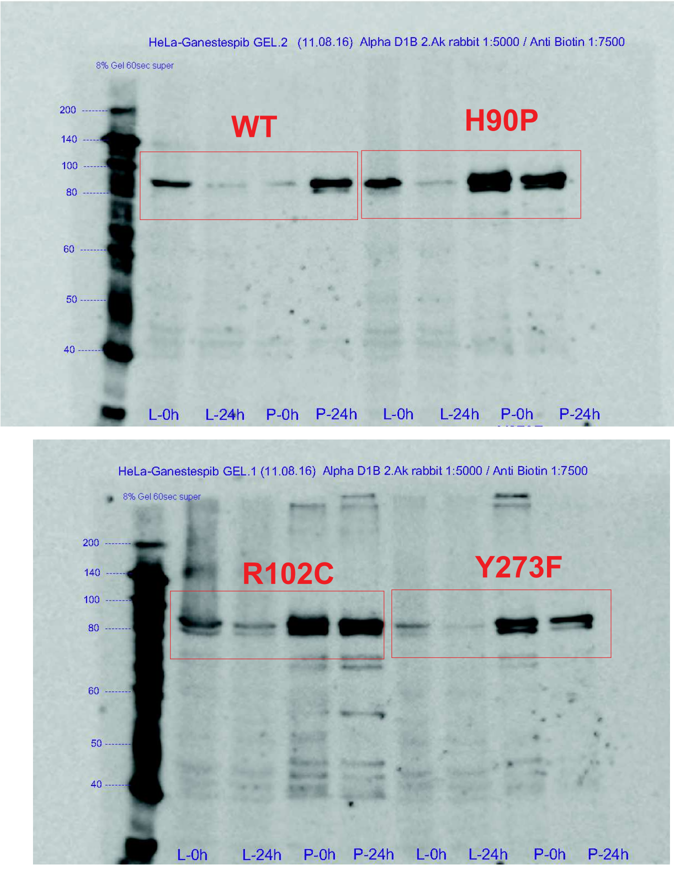

Fig. 7D

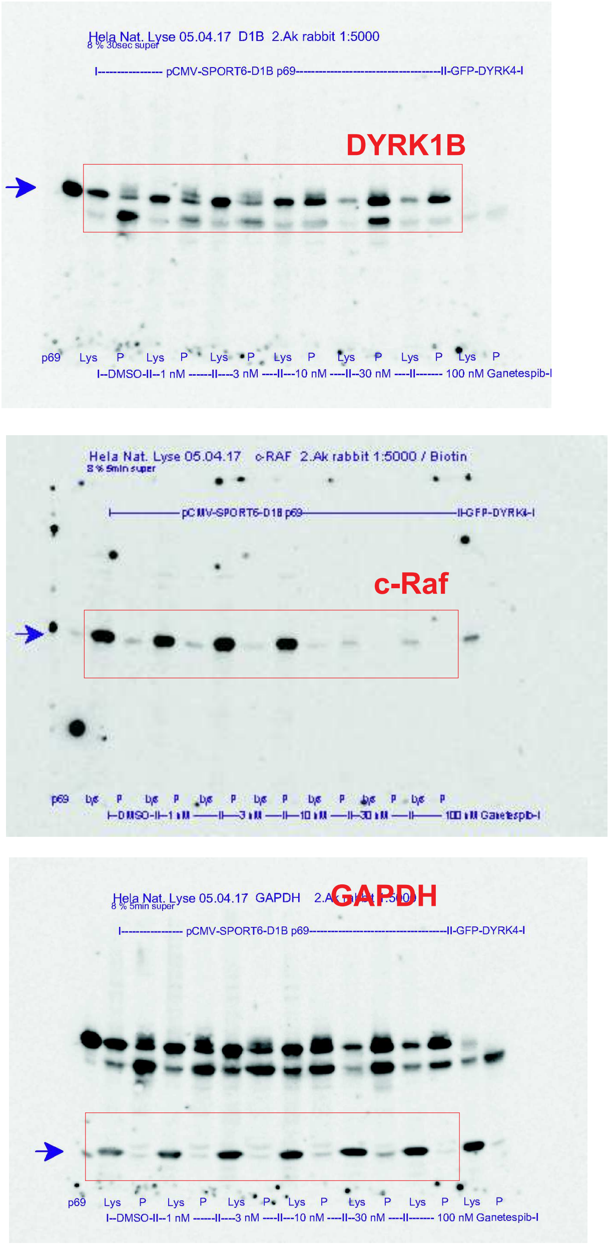

Fig. 7E

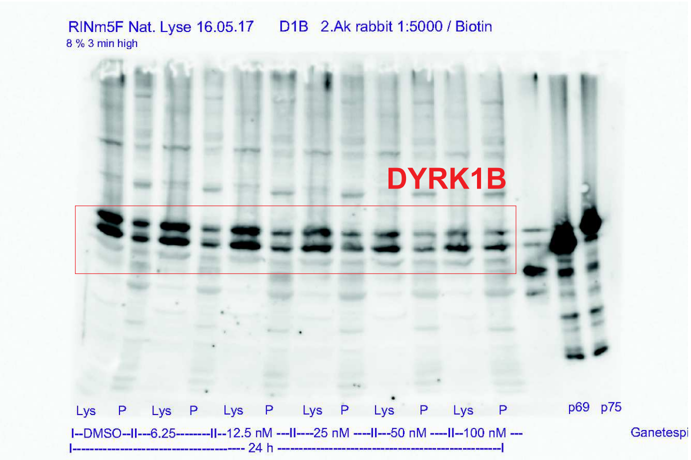

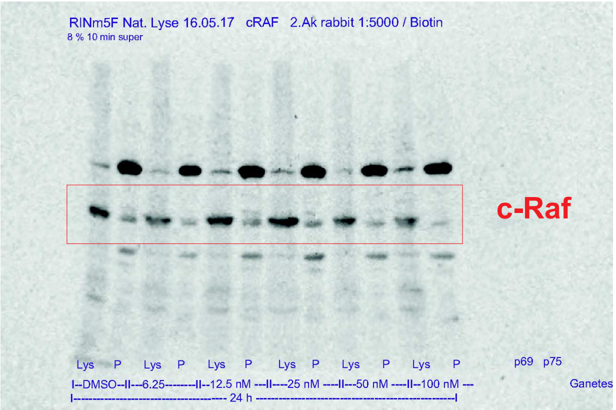

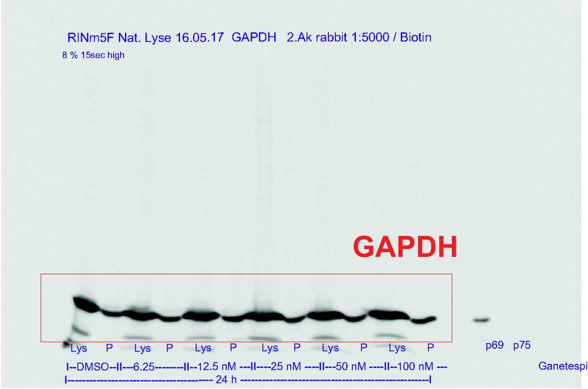

Supplement: Supplementary file 1 — Supplementary Information [file 41598_2017_6874_MOESM1_ESM.pdf]
